# Hotspots of Bacterial Pathogen Abundance and Exposure Risk in Soils of the Contiguous United States

**DOI:** 10.1029/2025GH001459

**Published:** 2025-12-11

**Authors:** Emily A. Matthews, Ying‐Xian Goh, Shannon L. Hepp, Jingqiu Liao, Ryan S. D. Calder

**Affiliations:** ^1^ Department of Civil and Environmental Engineering Virginia Tech Blacksburg VA USA; ^2^ Center for Emerging, Zoonotic, and Arthropod‐Borne Pathogens Virginia Tech Blacksburg VA USA; ^3^ Global Change Center Virginia Tech Blacksburg VA USA; ^4^ Department of Population Health Sciences Virginia Tech Blacksburg VA USA; ^5^ Department of Civil and Environmental Engineering Duke University Durham NC USA

**Keywords:** soilborne bacteria, outbreak, bacterial pathogen, environmental health, climate change, social vulnerability

## Abstract

Soils are reservoirs of pathogenic bacteria that cause human illness, particularly after mobilizing events such as extreme rain. Land‐use patterns (e.g., proximity to agriculture) and soil properties (e.g., moisture) are associated with abundance of individual pathogenic bacteria. However, there are major uncertainties in (a) the importance of local/regional land‐use decisions relative to overall natural variability of pathogenicity and (b) the correlations among pathogen abundance, climate‐linked physical processes increasing pathogen mobility, and the vulnerability of human receptors. This impairs identification of priority areas for outbreak surveillance, which has traditionally focused on food and water distribution networks, and the development of process‐based risk screening models. Here, we analyze a novel data set of 622 soil samples covering 42 of the 48 contiguous United States. We describe (a) the relationship between putative pathogenicity and natural and land‐use drivers and (b) how hotspots of putative pathogen abundance intersect with climate‐linked hazard of mobilization via fire, floods, wind, and fluvial transport, and the social vulnerability of local human populations. Variability in putative pathogenicity can be partially explained by known drivers, with natural variables having greater explanatory power than land‐use variables. Relative abundance of putative pathogens is generally higher in forested ecoregions, notably in the eastern and southeastern United States and in proximity to surface waters. Higher relative abundance of putative pathogens, climate risks promoting pathogen mobility, and a relatively vulnerable rural population intersect in the southeastern United States. Integrated sampling and modeling are needed to monitor and forecast health risks from soilborne pathogens.

## Plain Language Summary

Soil carries bacteria that cause illness, especially after storms and other events that cause people to be exposed (e.g., via flood water). Agriculture, certain soil properties, and other things are related to levels of harmful soil bacteria, but we do not know how important those factors are compared to overall variability, or which factors are most important. We also do not understand where highest abundance of the most harmful bacteria coincides with the risk factors for mobilization or with the most vulnerable human populations. In this study, we analyzed soil samples from across the United States to identify locations of the most harmful bacteria and the highest potential health risks (due to the vulnerability of people exposed and the possibility that bacteria would be mobilized). We found that natural factors like soil moisture were more important than land use decisions in explaining where harmful bacteria are most abundant. The eastern and southeastern U.S. stood out as areas where high levels of possible soilborne pathogens overlap with climate‐related risks and where many people may already be vulnerable due to poverty, rural living, or limited access to healthcare. This suggests that research and monitoring should focus on these regions.

## Key Points

- Natural properties predict putative pathogenicity of pristine soils better than land use, and variability is high even accounting for known drivers
- High pathogen potential intersects with climate‐linked mobilization risks and a vulnerable population in the southeastern United States
- Process‐based models and more intensive sampling are needed to disentangle natural soil reservoirs from anthropogenic sources in outbreaks

## 1 Introduction

Bacterial pathogens were responsible for roughly 13.7 million deaths and 304 million years of life lost worldwide in 2019 (GBD, 2022). Of all deaths and years of life lost attributable to bacterial pathogens, roughly half were attributable to bacteria with permanent or long‐term reservoirs in soils, notably *Escherichia coli*, *Listeria monocytogenes*, *Pseudomonas aeruginosa*, and *Enterobacter spp*. (Brennan et al., 2010; Cernava et al., 2019; GBD, 2022; Green et al., 1974). Soilborne bacterial pathogens (SBPs) can cause disease in humans via diverse transmission pathways, including direct ingestion of soil, mobilization of bacteria into drinking water resources and agricultural fields by precipitation, erosion, and animal movements (e.g., *Clostridium difficile*, *E*. *coli*) (Kotila et al., 2013; Liao, Bergholz, et al., 2021; Mukherjee et al., 2006; Ngure et al., 2013; Paulos et al., 2025) and atmospheric transport of bacterial spores into food (e.g., *C. botulinum*) (J. S. Palmer et al., 2019).

Outbreaks of bacterial disease are commonly classified by proximate mode of infection (e.g., foodborne, waterborne, person‐to‐person), which is the basis for public health surveillance and intervention infrastructure (CDC, 2024b; Lee & Yoon, 2021; Qiu et al., 2021; Quilliam et al., 2011). The outbreaks that are most likely to be detected are thus linked to large‐scale food, water, and social networks and where a high fraction of infected persons seek and receive medical attention (Bisht et al., 2021; Gibbons et al., 2014; Hrudey & Hrudey, 2007; Noppert & Zalla, 2021). Unreported cases may thus outnumber reported cases by factors of tens or hundreds depending on the pathogen and the surveillance infrastructure of the jurisdiction (Hall et al., 2008; Majowicz et al., 2005; Michel et al., 2000; Thomas et al., 2006). Soil reservoirs of pathogenic bacteria are however receiving increased attention for their role in contributing to human disease and serving as indicators for where increased surveillance may be warranted (Caimano et al., 2018; Paulos et al., 2025) in light of a large and growing literature linking disease outbreaks to the mobilization of pathogens from reservoirs in local environments (Auld et al., 2004; Austhof et al., 2024; Cann et al., 2013; Curriero et al., 2001).

Pathogenic exposures and exposure trends are particularly difficult to characterize among underserved rural populations. First, environmental exposure pathways are more heterogeneous and subject to less surveillance among rural than urban populations (e.g., drinking water wells serving individual families as opposed to public water systems serving whole cities); and, second, cultural, financial, and logistical barriers to healthcare make individuals in rural settings less likely to seek and/or receive medical attention than those in urban settings (Douthit et al., 2015; Institute of Medicine, 2006). Barriers to public health surveillance and intervention are compounded by higher pathogen abundance in rural and peri‐urban areas than in urban areas as a result of the greater proximity to unmanaged ecosystems (Chakraborty, 2013; M. Li et al., 2023; Pritchard, 2011). In the United States and elsewhere there are however major uncertainties as to where to focus surveillance efforts: there has been little analysis of how soil pathogenicity varies on a national scale or how this pathogenicity interacts with future climate risks or vulnerability of local populations.

The abundance and diversity of bacterial pathogens in soils are influenced by interactions with natural and anthropogenic physical, chemical, and biological processes (Samaddar et al., 2021). This includes the proximity and operational regime of agricultural sources (Figuerola et al., 2015); proximity of residential, industrial, or commercial development (Shaharoona et al., 2019; Strawn et al., 2013); soil texture, nutrients (e.g., carbon content) and pH (Hu et al., 1999; Nicol et al., 2008; Xia et al., 2020); and climatic variables, notably, precipitation (Ahmed et al., 2018; Angel et al., 2010; Wang et al., 2014). These correlations have been uncovered by methodologies that (a) correlate abundance of individual pathogens, and/or diversity of bacterial communities (e.g., *α*‐ and *β*‐ diversity) without reference to pathogenicity to environmental variables of interest (Chalmandrier et al., 2019; Prober et al., 2015; Tran et al., 2020) and (b) isolate the effect of individual environmental variables of interest by studying narrow sites with limited variability in other features (Gregory et al., 2019; Liao, Bergholz, et al., 2021; Oliva et al., 2023).

Risks from SBPs may be increasing as a result of climate change, driven by (a) the stimulating effect of warming temperatures on microbial pathogens and (b) enhanced transport of SBPs through the environment via more frequent floods, enhanced erosion, and other biophysical mechanisms (Boxall et al., 2009; Chakraborty, 2013; Dai et al., 2018; Delgado‐Baquerizo et al., 2020; Kelly et al., 2017; Z. Li & Fang, 2016; Mora et al., 2022; Pritchard, 2011; Rose et al., 2001; Singh et al., 2023; Wang et al., 2014). A small body of recent work has simulated the effects of expected temperatures (increased abundance) and precipitation (increased mobilization) on SBPs in receiving waters in diverse geographies, with mixed results. Jeon et al. (2019) suggest that both precipitation‐driven enhancements to transport and temperature‐driven increases in abundance are likely to increase fecal coliform concentrations in receiving waters in a Korean watershed. Cummins et al. (2015) suggest that the likely impact of climate exceeds that of land‐use changes in downstream transport of fecal coliforms in a watershed in southwestern Virginia, USA. By contrast, Sterk et al. (2016) find little effect of climate on runoff of *Campylobacter* and *Cryptosporidium* in the Netherlands. Divergent findings may arise from differences in site‐ and pathogen‐specific parameters across studies; differences in modeling strategy; or differences between pathogens in the net effects of climate on proliferation and/or transport. At present, available models differ greatly in their representation of microbial dynamics, microbe‐substrate interactions, and environmental transport processes (Bradford et al., 2013; Pachepsky et al., 2006).

More research is needed to understand present and future risks of SBPs, and an identification of high‐priority regions and settings is necessary to focus this work. Overall, however, there has been a lack of analysis of the relative importance of synergistic or antagonistic environmental drivers on soil pathogenicity, and major uncertainties over where these drivers combine to create hotspots of elevated pathogen risk. While the role of individual drivers on the abundance of SBPs of interest has been characterized in narrow geographic contexts, to our knowledge, there are no studies examining the significance of those relationships in the context of overall variability in pathogen abundance of relatively pristine soils (i.e., soil relatively unaffected by human activity). At the same time, it is poorly understood how variability in soil pathogenic potential interacts with susceptibility to climate‐induced enhancements to SBP mobility or the vulnerability of nearby human receptors (e.g., with higher burden of disease, less access to medical care, higher probability of exposure, etc.). Such analyses are needed to identify the highest‐priority regions and settings to focus public health surveillance and other research efforts such as the development of finer‐scale environmental models to quantify climate impacts on SBP exposure risks.

Here, we present a novel data set of soil putative bacterial pathogens covering the contiguous United States and analysis of patterns of tentative human pathogen distribution, relationships with diverse environmental variables, and elevated present‐day and future exposure risk. We identify priority areas for future research and surveillance based on the intersection of pathogenic potential of soil bacteria, the likelihood for present and future environmental mobilization, and the vulnerability of nearby populations. Whereas previous analysis has characterized the role of environmental variables on microbial diversity and/or pathogenicity in narrow geographic settings, we describe variability in pathogenic potential over the contiguous United States, describing drivers of variability nationally, and identifying where heightened surveillance and research is needed.

## 2 Methods

### 2.1 Microbial Sampling and Sequencing

This study used existing 16S rRNA amplicon sequencing data, which characterize bacterial communities, for 622 soil samples collected from natural environments across the contiguous United States. The methods for sample collection and16S rRNA amplicon sequencing were detailed in Liao, Guo, et al. (2021) and Liao et al. (2023) respectively. In brief, topsoil (0–20 cm) was collected at each sampling site following a standard protocol. Total DNA was extracted from soil samples using QIAGEN DNeasy PowerSoil Pro Kits. The V4 region of the 16S rRNA gene was amplified from total DNA and sequenced on a MiSeq using a 2 × 250 bp paired‐end read run. Raw reads were denoised using DADA2 and classified into amplicon sequence variants (ASVs) using QIIME2 (Beiko et al., 2018; Callahan et al., 2016).

**Figure 1:**
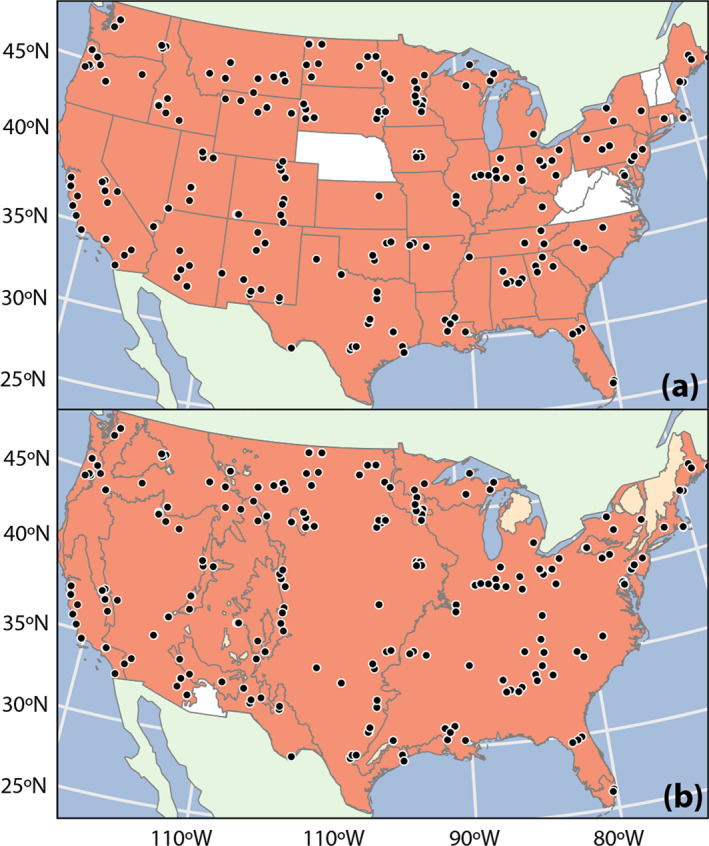
Map of the contiguous United States showing 622 sampling locations across (a) states and (b) ecoregions. States and ecoregions with sampling locations are shaded in dark orange and those with no sampling locations are white. Non‐contiguous sections of ecoregions with sampling points are shaded in lighter orange in (b) if those noncontiguous sections do not have sampling points. Projection: Albers CONUS.

The 622 samples cover 42 of the 48 contiguous U.S. states (Figure 1a). The Commission for Environmental Cooperation divides North America into ecoregions based on similarities in physiographic features such as geology, soil type, climate and hydrologic patterns, and plant and wildlife distributions (CEC, 1997; U.S. EPA, 2023). Of the ten level‐one ecoregions in the contiguous United States, samples cover all except the small portion of the Southern Semiarid Highlands that extends into Arizona (Figure 1b). Maps of ecoregions at different levels of detail are available from U.S. EPA (2023) or on request from the authors. Ecoregion‐ and state‐specific results are discussed in Section 3.1.

### 2.2 Bacterial Pathogen Detection and Classification

Putative bacterial pathogens infecting humans were identified by mapping sequences of ASVs to the 16S pathogen database for human pathogens provided by 16SPIP, a comprehensive analysis pipeline for rapid pathogen detection based on 16S rRNA amplicon sequencing (Miao et al., 2017), using the nucleotide‐nucleotide basic local alignment search tool (BLASTn) (Ye et al., 2006). An E‐value of 0.01 and no restrictions on percent identity were used in the BLASTn searches. The hit with the highest bit‐score was chosen for each ASV. ASVs with a sequence coverage of >80% and identity of >97% were classified as putative human pathogens.

Pathogens were classified into three risk groups (RG), RG1 to RG3, based on their potential to cause illness in healthy individuals, following NIH (2024) and ABSA (2024). A fourth risk group, RG4, contains only viruses and therefore is not relevant to this analysis. RG1 agents are not associated with disease in healthy adults and include *Bacillus subtilis*. RG2 agents are associated with disease for which treatments are readily available including *B*. *anthracis*, *E*. *coli*, and *C*. *botulinum*. RG3 agents are associated with serious or lethal disease with lesser availability of preventative or therapeutic interventions and include *Mycobacterium tuberculosis* (causing tuberculosis). A full list of agents included in RG1, RG2, and RG3 Risk Groups identified in our data set is included in Table S1 in Supporting Information S1. This study focuses primarily on observations of RG2 and RG3 given their higher potential for pathogenicity and adverse human health impacts.

### 2.3 Environmental Data

We created a database of natural and land‐use variables georeferenced to soil sampling locations described in Section 2.1. This database is used to characterize the biophysical drivers of relative abundance of putative pathogens in soils across the contiguous U.S. using the statistical approach described in Section 2.6. Table S2 in Supporting Information S1 summarizes all variables used in this analysis and provides links to their original provenance. A brief description of these variables is provided below.

#### 2.3.1 Natural Environment Variables

Natural soil property data used in this study include soil concentrations of: total nitrogen, total carbon, organic matter, aluminum, calcium, copper, iron, potassium, magnesium, manganese, molybdenum, sodium, phosphorus, sulfur, and zinc; moisture content; and pH previously reported in Liao, Guo, et al. (2021). Climatic variables included are: average precipitation; average wind speed; maximum daily average temperature; and minimum daily average temperature over the period 1985–2011 (NOAA, 2025).

Proximity to surface water is included and was calculated by comparing the spatial coordinates of each sampling location to the “Area” feature of the medium‐resolution (1:24,000 scale) National Hydrography Data set (NHD) (USGS, 2023). For each sampling location, proximity was calculated as the shortest distance to a vertex of a line representing a streambank in the NHD. This analysis was completed in QGIS v. 3.16.14 (QGIS.org, 2022) using Virginia Tech's Advanced Research Computing (ARC) resources.

Geolocation variables (latitude, longitude, and elevation, ecoregion, and state) are included to account in certain model formulations to measure the remaining uncontrolled spatial correlations.

#### 2.3.2 Land‐Use Variables

Land‐use variables considered as potential drivers include 10‐km‐radius averages of the following land use categories previously reported in Liao, Guo, et al. (2021) and originally derived from the National Land Cover Database (USGS, 2024): fraction as open water; barren; forest; shrubland; grassland; cropland; pasture; wetland; and developed open space categorized as >20% and <20% impervious cover. These were supplemented with 10‐km‐radius facility counts from the National Pollutant Discharge and Elimination System (NPDES) (U.S. EPA, 2025c); the National Priorities List (Superfund, NPL) (U.S. EPA, 2025a); sites identified in the Risk Management Programs (RMP) Facility Registry System (U.S. EPA, 2025b); and Treatment, Storage, and Disposal Facilities (TSDF) (U.S. EPA, 2025b). Alternative approaches such as discharge‐ or toxicity‐ weighted counts were considered but excluded because of data missingness and insignificant impact on model performance.

NPL, RMP, and TSDF were included to serve as a proxy for potential major sources of pathogenic bacteria in the local environment. NPL covers federally listed Superfund sites such as contaminated industrial facilities, landfills, and sludge lagoons; RMP covers facilities that manufacture, use, or store hazardous chemicals including food processors and water treatment plants; and TSDF covers hazardous‐waste treatment, storage, and disposal units, such landfills. The 10‐km radius was retained for consistency with Liao, Guo, et al. (2021); results indicate that NPL, RMP, and TSDF variables have little explanatory power across model formulations (Section 3.2); this did not change with any radius explored, and so 10 km was left as the default.

### 2.4 Flood, Wildfire, and Wind Mobilization Hazards

Flood, wildfire, and wind are natural hazards that can mobilize soil pathogens to downstream receptors either directly (flood, wind) or indirectly through erosional processes (fire). We mapped these hazards across the contiguous United States and co‐located each with the putative pathogenicity of soil samples (Section 2.2) to identify areas where high potential for climate‐linked transport coincides with high microbiological risk.

The First Street Foundation (FSF) provides a national atlas of property‐specific flood, wildfire, and wind risk with data aggregated to the Census block group level available for public download. For each block group, the number of properties rated at “Risk Factors” ranging from 1 (least risk) to 10 (highest risk) is reported (FSF, 2024). FSF Risk Factors present the advantage of intercomparability between hazards, national coverage, public availability, and ease of integration into a large data set (as compared to, e.g., data from FEMA flood risk maps).

For each mobilization hazard (flood, wildfire, and wind), we defined a block‐group‐level Risk Factor score calculated as the average of Risk Factor levels weighted by the number of properties at each risk level. That is, the Score (*S*) in block group *j* is defined as $S_{j}=\sum_{i}\left(R_{i}\times n_{i,j}\right)/\sum_{i}n_{i,j}$ where *R*_*i*,_ is Risk Factor from i∈{1..10}, and $n_{i,j}$ is the number of properties at each Risk Factor level in that census block. This treats the ordinal Risk Factors as interval data and uses the distribution of Risk Factor levels across properties in the Census block as a proxy for the relative magnitude of that hazard in the Census block as a whole. The distribution of Risk Factor scores associated with our samples closely matches the distribution of Risk Factors scores across all Census blocks in the United States. Figures S1a–S1c in Supporting Information S1 plot histograms of Risk Factor scores for flood, wind, and fire, respectively. Tracts at low wind risk (0–1 out of 10) are somewhat oversampled (70% of samples compared to 50% of Census tracts) and low fire risk (0–1 out of 10) are somewhat undersampled (36% of samples compared to 58% of Census tracts). Scores are approximately lognormally distributed for all hazards, both for the block groups in which soil samples were taken and more broadly across the United States.

In addition to considering each hazard type individually, we also consider a composite score as the product of all hazard scores. We calculate this using a product‐based rather than a sum‐based approach to better identify potential areas of extreme risk driven by interacting transport mechanisms. This is of particular interest from the perspective of identifying priority areas for more granular research, particularly on interacting transport mechanisms, which is the focus of the present analysis. However, it presents the disadvantage of downweighting the importance of areas where one or more mobilizing hazards is very low. Furthermore, the way in which mobilizing hazards interact is likely to be site‐specific and unlikely to be the same across the U.S. We therefore discuss both results deriving from the composite mobilizing hazard and from individual hazards.

### 2.5 Drinking Water Receptor Vulnerability

We evaluated putative pathogenicity in areas served and unserved by public water systems using the database developed by Buchwald et al. (2022). Previous analysis has identified that environments at the urban/rural fringe may be at higher risk for microbial exposures due to both land‐use patterns (e.g., proximity to agriculture) and exposure patterns (e.g., more direct contact of residents with soil due to outdoor activities) (Angel et al., 2010; M. Li et al., 2023). Because these environments are often also transition points from public water systems to more vulnerable private wells, we tested the hypothesis that relative abundance of putative pathogens was higher in areas unserved by public water systems. We also identified areas of high putative pathogenicity that intersect with well water usage as part of the broader vulnerability assessment.

### 2.6 Sociodemographic Data

The U.S. Centers for Disease Control and Prevention compile the Social Vulnerability Index (SVI), which quantifies demographic and socioeconomic characteristics of communities associated with vulnerability to environmental hazards and natural and anthropogenic stressors (CDC, 2024a). The SVI is calculated at the county level using 16 variables grouped into four “themes”: (a) socioeconomic (e.g., unemployment rate); (b) household characteristic (e.g., age and disability status); (c) racial and ethnic minority status; and (d) housing type and transportation (e.g., mobile homes, lack of vehicles). These variables are then used to calculate an overall vulnerability score. We use Census tract‐level SVI data to understand where high present or potential future risks intersect with high vulnerability to identify geographies where heightened surveillance or outreach may be warranted.

### 2.7 Statistical Analysis and Visualizations

Statistical analysis and visualizations were carried out in RStudio version 2024.12.1 + 563 running R version 4.4.3(R Foundation for Statistical Computing, 2022; RStudio Team, 2022). Figures were edited in Adobe Illustrator version 28.5 (Adobe, 2024). R packages used to make plots include ggplot2, maps, rnaturalearth, rnaturalearthdata, viridis, patchwork, and RColorBrewer (Becker et al., 2023; Garnier et al., 2024; Hunter, 2007; Massicotte et al., 2023; Neuwirth, 2022; Pedersen, 2024; South et al., 2024; Wickham et al., 2024). Python packages include Matplotlib v.1.2.1 (Hunter, 2007). Spatial analysis and visualizations use the Albers CONUS projection.

#### 2.7.1 Putative Pathogen Relative Abundance and Drivers

The Shannon‐Wiener diversity index of SBPs was computed for each sample using the Python scikitbio library (Rideout et al., 2023). The association between Shannon‐Wiener diversity index and relative abundance of putative SBPs was assessed using Spearman correlation. A Spearman's rank correlation analysis was also performed to evaluate associations between the Shannon‐Wiener diversity/relative abundance of SBPs and each environmental variable for all RG combined and each RG individually. A Benjamini‐Hochberg (BH) false discovery rate (FDR) adjustment was applied to account for multiple testing. Variables with an FDR‐corrected *p* value < 0.05 were considered significant.

Spatial autocorrelation was assessed with Moran's test using packages sp and spdep (Bivand et al., 2024; Pebesma et al., 2024). This test evaluates the null hypothesis that the attribute of interest (e.g., RG1, RG2, etc.) is randomly distributed across features defined with reference to the distances between each (i.e., sampling locations separated by great‐circle distances) and provides a measure of clustering or dispersion. Moran's statistic and *p*‐values were calculated individually for all measures of putative pathogenicity (RG1, RG2, RG3, and Shannon‐Wiener diversity index) by calculating distance matrices for all points within 10° latitude and longitude of all other points.

Random forest models to explain drivers of putative pathogen relative abundance were developed using the R package randomForest (Liaw et al., 2022). This is a machine learning method that classifies data points according to the magnitude of the outcome variable of interest (e.g., RG1, RG2, etc.), creating parsimonious “trees” of predictor variables branching at cutoff values. The final model is a composite of all trees, each calculated using a different random subset of data. Random forests were trained on a random selection of 85% of the data points (528 out of 622) and evaluated against the remaining 15%. Models were evaluated both using all predictor variables and a smaller data set excluding variables featuring high multicollinearity (variance inflation factor >10). Model performance was evaluated by comparing values of the reserved 15% of samples to the values predicted under the classification model and calculating the fraction of variance in the measured values explained by the modeled values (*R* ^2^).

Classification models like random forests are highly suited to uncovering drivers of complex, multifactorial environmental processes (Bourne et al., 2025; Brokamp et al., 2018; Shi et al., 2021). First, they do not make any assumptions about the structure of data (e.g., normality, homoscedastisity, etc.), which are rarely respected by environmental data. Second, they do not assume a model shape (e.g., linearity), making them well suited to environmental processes which are frequently nonlinear and involve complex and poorly understood interactions between variables (Calder et al., 2023). This includes notably microbiological dynamics in the natural environment, which feature poorly understood and potentially site‐specific relationships among variables, which limits the adequacy and interpretability of classical statistical investigations (Lynch & Shaman, 2024). These models however require careful analysis of mechanistic plausibility to develop confidence that the model structure retained is likely to reflect underlying physical processes. Therefore, we also undertake an analysis of the mechanistic interpretation of the results of the random forest model uncovered, for example, by examining direction and strength of association between predictor and response variables in the context of previous scientific investigations.

#### 2.7.2 Composite Scores to Identify Priority Areas

To screen regions for intersections of (a) high relative abundance of putative pathogens; (b) high relative risk of physical hazards that may mobilize pathogens; and (c) high vulnerability of human receptors to those risks, we multiplied (a) relative abundance of putative pathogens (individually by risk group) with relative physical hazard risk (individually and for all hazards combined); and (b) relative putative pathogen abundance (individually by risk group) with the SVI (individual SVI axes and the total SVI). Consistent with our approach for characterizing cumulative hazards described in Section 2.4, we adopted a product‐based approach to identify areas of high risk. Putative pathogen/physical hazard and putative pathogen/SVI intersection areas were compared qualitatively.

## 3 Results and Discussion

Here we describe observations about the spatial distribution of putative pathogens across the contiguous U.S. (Section 3.1); the extent to which relative abundance of putative pathogens is explained by soil properties and local land‐use characteristics (Section 3.2); correlations of relative abundance of putative pathogens with features that may promote mobilization to human receptors (Section 3.3); the social vulnerability of populations in proximity to hotspots of putative pathogenicity (Section 3.4); and the vulnerability of well‐water users specifically (Section 3.5). In brief, relative abundance of putative pathogens (from both RG2 and RG3 groups) is clustered but broadly distributed, including hotspots in the southeastern and midwestern U.S. and in California. Natural soil properties explained more variability in relative abundance of putative pathogens than did land‐use variables. However, this elevated risk intersects with both risk of climate‐linked mobilization and a relatively socially vulnerable rural population in the southeastern U.S. No correlation with public drinking water versus well coverage was observed.

### 3.1 Spatial Distribution of Putative Human Bacterial Pathogens in Soils Across the U.S.

A total of 293 ASVs were identified as putative human bacterial pathogens, representing 40 species. These 40 species were grouped into RGs, with nine being classified as RG1 (e.g., *Bacillus subtilis*, *Lactococcus lactis*, and *Microbacterium oxydans*), 28 as RG2 (e.g., *Bacillus cereus*, *Bacillus thuringiensis*, *Clostridium perfringens*, *Escherichia coli*, *Acinetobacter baumannii*, and *Staphylococcus saprophyticus*) and three as RG3, including *Bartonella elizabethae*, *Brucella melitensis*, and *Mycobacterium tuberculosis* (Table S2 in Supporting Information S1). RG2 showed the highest mean relative abundance across samples, followed by RG1 (Figure S2a in Supporting Information S1). The overall putative pathogen diversity indicated by Shannon‐Wiener index ranged between 0 and 0.97 with mean ± sd of 0.08 ± 0.07 and is significantly highly correlated with the relative abundance of putative pathogens (Spearman *ρ* = 0.99, *p* < 0.001; Figure S2b in Supporting Information S1).

Moran's test was significant (*p* < 0.001) for RG1, RG2, RG3, and the Shannon‐Wiener index of SBPs with negative I‐statistic, pointing to spatial clustering of putative SBPs. In particular, several areas in the western and the midwestern U.S. exhibit high diversity and abundance. Hotspots of RG1 were found in Montana and North Dakota. RG2 hotspots covered a larger spatial scale, including in the eastern and western coastal areas and Midwest. RG3 was mostly identified in the eastern U.S., with a low relative abundance and many nondetects. RG1, RG2, and RG3 had nondetect rates of 12%, 3%, and 76% respectively. Figures S2c–S2g in Supporting Information S1 illustrates this spatial distribution on maps. Figures S3 and S4 in Supporting Information S1 represent the distribution of relative abundance of RG1, RG2, and RG3 by state and ecoregion, respectively. Notably for RG2 and RG3, the Eastern Temperate Forest zone emerges as the ecoregion with highest mean relative abundance. The Tropical Wet Forests zone also has higher mean RG3 values but (in the United States) is confined to the southern tip of Florida. This is consistent with previous observations that pathogenic bacteria such as listeria are favored in soils that are relatively moist and rich in organic matter, and which provide heterogeneous microhabitats, such as those in forested ecoregions (Angel et al., 2010; Liao, Guo, et al., 2021). These results indicate large spatial heterogeneity in distribution of soil bacteria varying in virulence potential across the contiguous U.S. Recent investigations have identified hotspots of pathogenicity on a local or regional spatial scale (M. Li et al., 2023, 2025), but to our knowledge, this is the first to identify similar clustering on a larger spatial scale. Natural and anthropogenic drivers of putative pathogen abundance are described in Section 3.2. Overlap with climate‐linked physical risk factors for pathogen mobilization is described in Section 3.3, and with receptor vulnerability in Section 3.4.

### 3.2 Drivers of Putative Pathogen Abundance Across the U.S.

**Figure 2:**
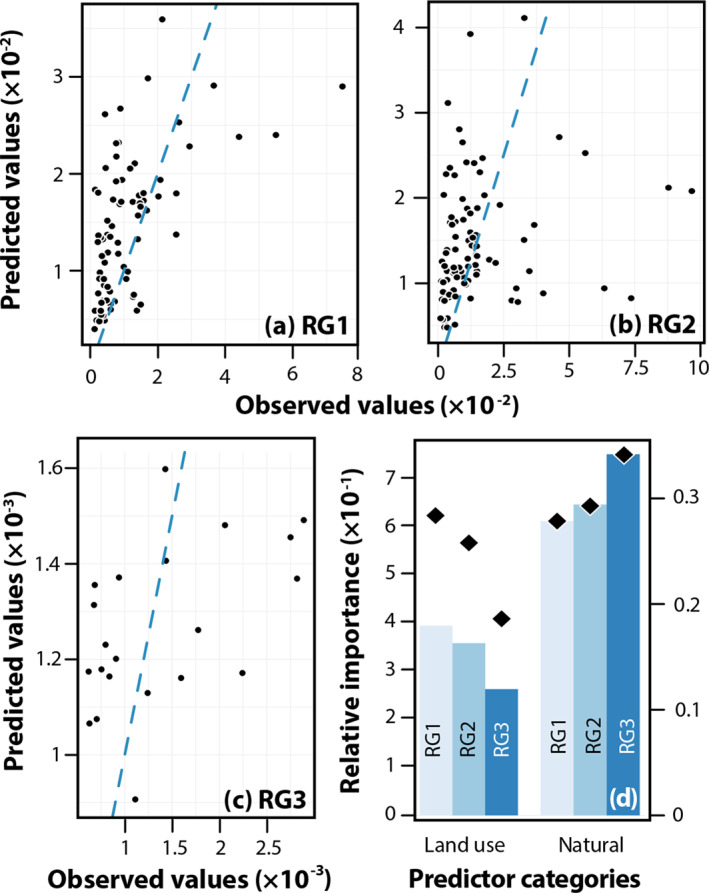
Random forest predictions versus observations for RG1 (a), RG2 (b), and RG3 (c) on test data (15% of data points not used for model development). Hatched blue line is 1:1 relationship between model and observation. Pane (d) plots importance (sum of increase in node purity) of predictors within “land use” and “natural” groups of predictors for all predictors combined (bars, left axis) and normalized by the number of variables within each group (diamonds, right axis).

A random forest classification model developed on a random subset of 85% of the data demonstrated moderate predictive power for risk groups RG1, RG2, and RG3 abundance among the remaining 15% of samples across the United States (Figure 2), excluding values below the 1st and above the 99th percentile. This model used the suite of mechanistically interpretable natural and land‐use variables described in Sections 2.3.1 and 2.3.2; latitude, longitude, state, and ecoregion were not considered in this model but were retained to explore the predictive power associated with residual spatially/environmentally correlated drivers not already modeled explicitly. Variance in training RG values explained by this mechanistically interpretable random forest models is 18% (RG1), 14% (RG2), and 6% (RG3). Notably, predictive power of environmental variables declines with increasing risk group but also with increasing putative pathogen relative abundance *within each* risk group. Thus, variability on a national scale is only moderately explained by associations with known natural and land‐use drivers, and predictive power declines for more relevant pathogen groups.

Adding state, ecoregion, latitude, and longitude to the model to account for other spatial effects increased the fraction of variance explained from 21% to 23% for RG2 and from 16% to 21% for RG3 but did not improve performance for RG1. Excluding variables based on multicollinearity (either across predictor categories pooled together or within predictor categories individually) did not improve model performance. All random forest model output is included in Tables S3–S5 in Supporting Information S1 (baseline model presented here for RG1, RG2, and RG3 outcomes, respectively); Tables S6–S8 in Supporting Information S1 (baseline model plus residual spatial variables); Tables S9–S11 in Supporting Information S1 (variables dropped due to multicollinearity across all predictor categories); and Tables S12–S14 in Supporting Information S1 (variables dropped due to multicollinearity within predictor categories).

Across all risk groups, land use variables (those most responsive to short‐term, local decision‐making such as developed fraction, number of nearby NPL sites, etc.) were less significant than natural variables, both overall and when normalized for the number of variables classified as “land use” (development fraction, etc.) and “natural” (metals, moisture, etc.) as shown in Figure 2d. Importance of anthropogenic land use predictors relative to natural predictors declined monotonically with increasing risk group. Natural variables related to temperature and moisture (e.g., soil moisture, distance to stream), metals, and organic matter content are consistently among the most significant variables for all risk groups. Their importance relative to land‐use variables is higher among RG3 than among RG1 and RG2. Very anthropogenically influenced predictors such as number of RMP and TSDF sites were of consistently low importance. The importance of individual predictors across model formulations is tabulated in Table S3–S14 in Supporting Information S1.

As described in Section 3.1, the southeastern U.S. emerges as a region with relatively high relative abundance of putative pathogens in soil; it is also a highly diverse agricultural environment, presenting many pathogenic point sources (Asseng et al., 2013). In the context of overall national variability, however, higher agricultural density did not emerge as a significant predictor of higher soil pathogenicity, possibly because samples were not collected after/down‐gradient from acute mobilizing events. Nevertheless, these sources may add to the overall risk level in the southeastern U.S. A more focused process‐based analysis would be necessary to understand the additive risk of latent reservoirs in pristine soils (studied here) and risks posed by agricultural sources that can be mobilized by heavy rains and other events.

**Figure 3:**
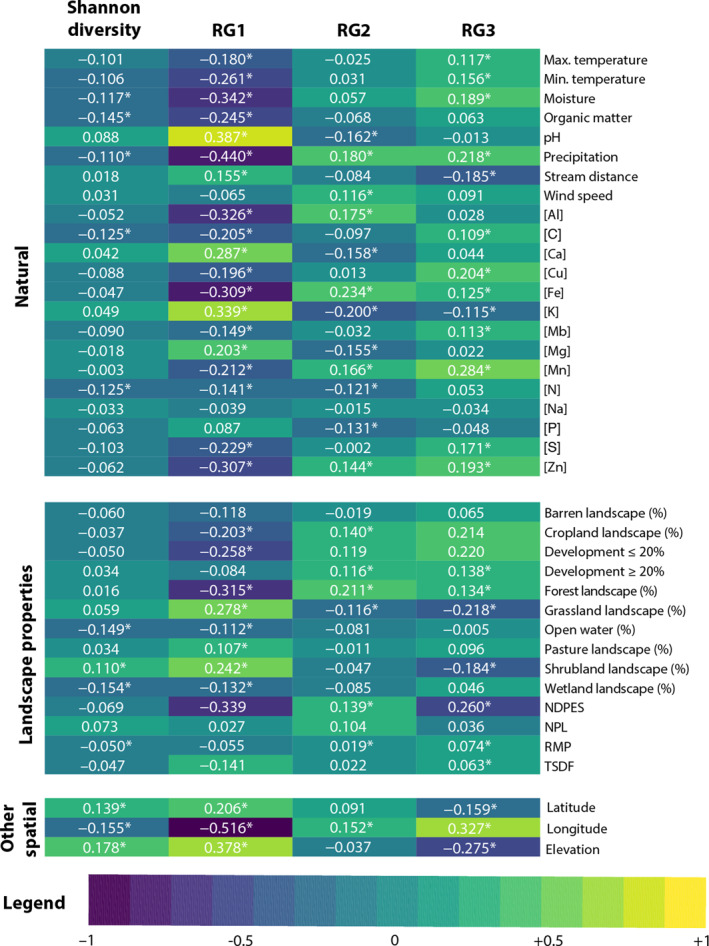
Correlations among environmental variables and the diversity and relative abundance of putative human pathogens in soils, including RG1, RG2, and RG3. * denotes *p* < 0.05 (adjusted for multiple comparisons).

Findings from random forest modeling are broadly consistent with one‐way correlations calculated using the Spearman coefficient with significance adjusted for multiple comparisons (Figure 3). Of note, the effects of many environmental variables on RG1 appear to be opposite on RG2 and RG3. For example, mean precipitation and soil moisture are significantly negatively correlated with Shannon‐Wiener diversity and RG1 and positively correlated with RG2 (significant for precipitation and soil moisture) and RG3 (significant for both precipitation and soil moisture). This is consistent with previous investigations that have found that, generally, the environmental conditions favoring pathogenic bacteria may lead them to outcompete nonpathogenic bacteria (Bengtsson‐Palme et al., 2017; Trinh et al., 2018) and that pathogenic bacteria are more viable in soils with lower overall diversity (van Elsas et al., 2012).

Taken as a whole, these findings suggest that, in the range of relatively pristine environments sampled here, the role of identifiable (particularly anthropogenic) drivers of microbial abundance declines relative to natural variability with increasing soil pathogenicity. This is suggested by model performance that declines (a) within risk groups for increasing relative abundance and (b) across risk groups for increasing pathogenic potential; and the relatively low importance of land use predictors that declines with increasing risk group. The importance of individual drivers uncovered here is consistent with previous findings, for example, the role of soil moisture and hydrographic features in proliferation and transport of pathogenic bacteria (Aung et al., 2018; Jiang et al., 2021; Mallin et al., 2000). This national scale analysis however reveals that such “natural” features are likely to drive overall variability in the abundance of the most pathogenic bacteria outside of the immediate environment of major environmental sources (i.e., on a regional basis), despite the role of major local role of sources such as agricultural facilities (Caimano et al., 2018; Figuerola et al., 2015; Shaharoona et al., 2019; Strawn et al., 2013).

### 3.3 Physical Risk Factors

Here, we describe how relative abundance of putative pathogens, particularly in RG2 and RG3, correlate with features that present potential exposure pathways. This includes surface water proximity (Section 3.3.1), which was also used as a predictor of relative abundance (Section 3.2), and risk scores for fire, flood, and wind (Section 3.3.2).

#### 3.3.1 Surface Water Proximity

Proximity to surface water was revealed to be a significant predictor of relative abundance of putative pathogens, potentially due the role of closely related properties such as soil moisture and/or the role of the hydrographic network in pathogen transport (Section 3.2). This association is also relevant from the perspective of hazard analysis, as near‐stream environments are more vulnerable to mobilizing events such as floods and erosion.

**Figure 4:**
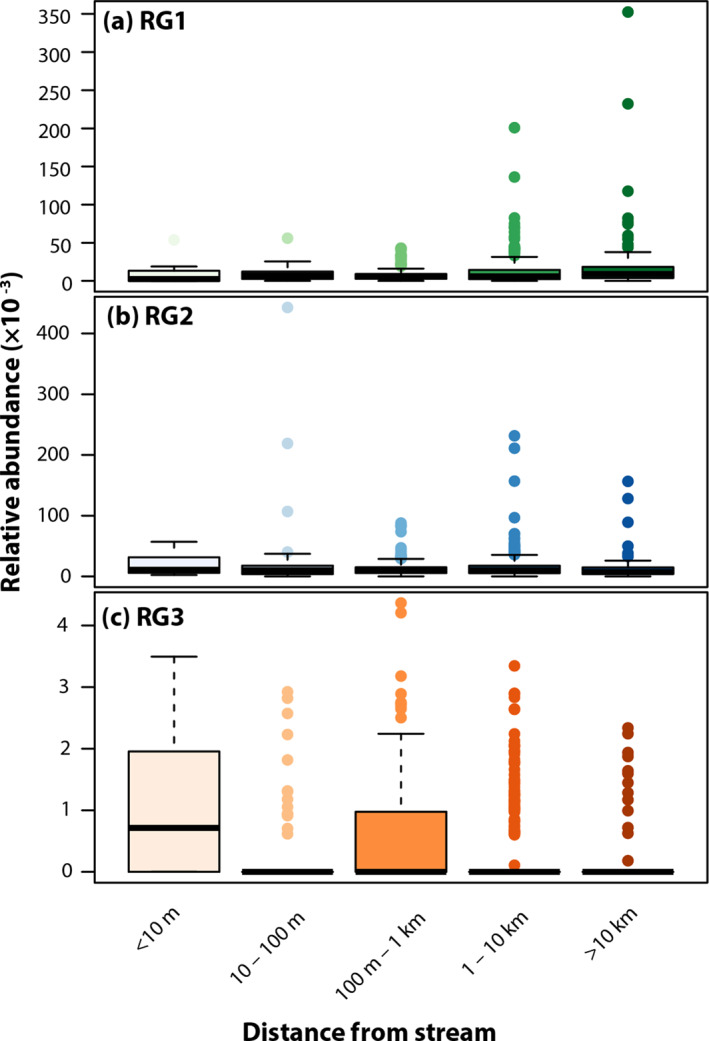
Relative abundance of putative pathogens in risk group (RG) 1 (a), RG2 (b), and RG3 (c) by distance of sampling station to nearest body of water identified from the NHD.

Mean abundance of RG3 and RG2 putative pathogens was 3.3x and 1.2x higher, respectively, among samples collected ≤10 m from surface water than among other samples (Figure 4). This is consistent with previous investigations on much smaller spatial scales, which have found that aquatic sediments and beach sands are local reservoirs of pathogenic bacteria and that abundance declines exponentially with distance from surface waters (Byappanahalli et al., 2003; Desmarais et al., 2002; Pachepsky & Shelton, 2011). It is however notable that this effect is as pronounced in a national data set spanning ecological regions, levels of development, and ranges of proximity to other environmental sources. Consistent with observations described earlier that many features have opposite correlations among RG1 versus RG2 and RG3, RG1 relative abundance is lower in proximity to surface water than elsewhere (e.g., 0.9x within 10 m as compared to others).

#### 3.3.2 Fire, Flood, and Wind Risk Scores

**Figure 5:**
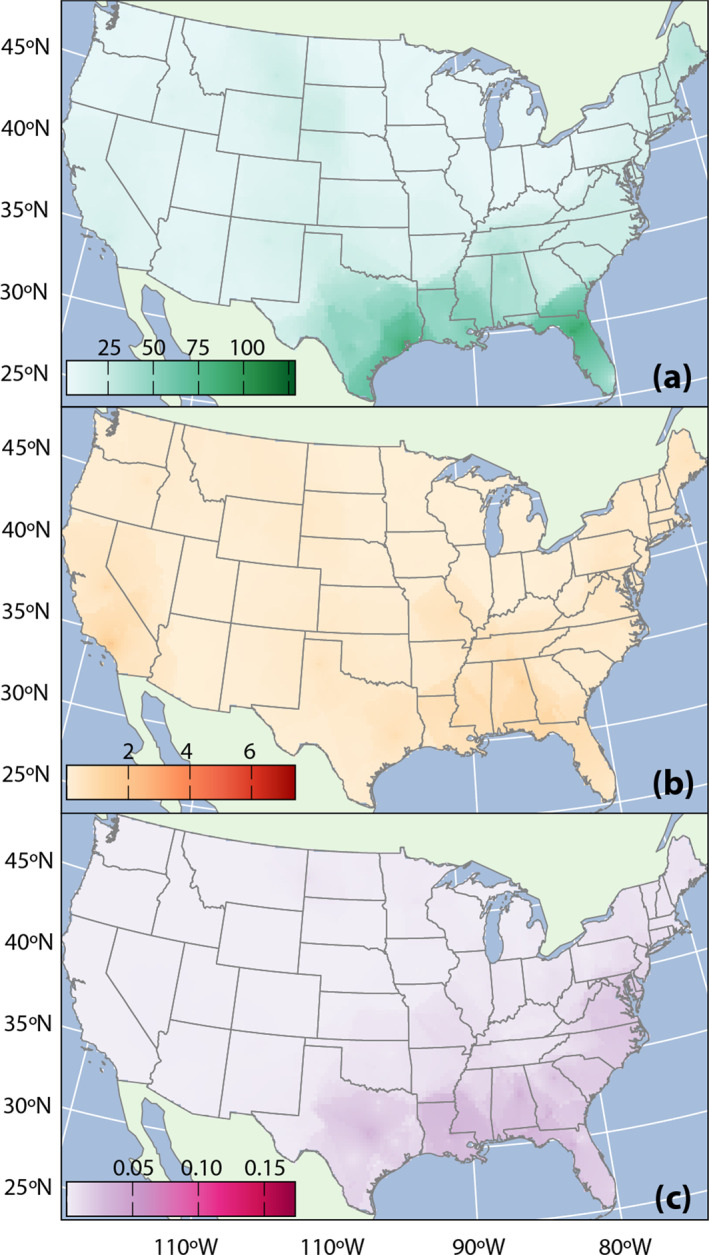
(a) Composite risk score (product of fire, flood, and wind) for all Census tracts; and composite risk score multiplied by relative abundance of (b) RG2; and (c) RG3. Projection: Albers CONUS.

Areas of elevated RG2 and RG3 abundance intersect with high fire, flood, and wind risk to create an identifiable region of interest in the southeastern United States. High physical risks around the Gulf coast combine with elevated RG2 and RG3 abundance in the eastern United States more generally to create an area of increased risk of SBP mobility and potential for human exposures. Figure 5 plots a heatmap of the composite risk score for fire, flood, and wind hazards (a) as well as the product of that composite score with the relative abundance of RG2 (b) and RG3 (c). Combined physical‐RG3 hazards are more localized in the southeastern United States than RG2. Results are similar when comparing the intersection of RG2 and RG3 abundance with individual fire, flood, and wind risks. However, high fire risk on the California coast coincides with an area of elevated RG2 abundance in southwestern California. Likewise, flood risks intersect with higher RG2 abundance in the midwestern and northeastern U.S. Figure S5 in Supporting Information S1 presents risk maps for fire, flood, and wind, and Figure S6 in Supporting Information S1 presents heatmaps for the intersection of RG2 and RG3 abundance with individual flood, fire, and wind risk scores.

We note that many areas of higher RG2 and RG3 abundance were in parts of the country with relatively lower flood, fire, and wind risk, notably, in the Midwest. This is reflected by the broad spatial distribution of intermediate values for combined physical‐RG2 hazard shown in Figure 5b. In general, correlations between relative abundance of soil putative pathogens and physical hazards (individual and composite) were weak. This is consistent with results presented in Sections 3.1 and 3.2 that pointed to high national variability in pathogenic potential that remains after accounting for hypothesized land‐use, soil property, and climatic drivers, and residual spatial clustering.

### 3.4 Socioeconomic Vulnerability

Elevated relative abundance of RG3 intersects markedly with socioeconomic vulnerability in the southeastern United States (Figure 5), while spatial interactions are less pronounced for RG2. For example, interactions between RG2 and SVI are greater in parts of California and the Midwest. SVI maps by theme (or and combined across themes) are available from U.S. CDC (2024). Figure S7 in Supporting Information S1 plots how SVI percentiles within themes intersect with relative abundance of RG2 and RG3.

High social vulnerability compounds risks of exposure and transport described earlier because the human populations in these areas disproportionately lack resources to seek medical care; have high burden of disease, making them more susceptible to the effects of bacterial infection; lack resources to evacuate in the event of pathogen‐mobilizing extreme weather events; and are more likely to come into contact with mobilized environmental media (e.g., flood waters, soil, dust) due to lower quality housing and higher prevalence of outdoor work.

Previous investigations have shown that individuals in rural environments are more vulnerable to soilborne pathogen exposure because of (a) increased proximity to certain sources to agricultural facilities and (b) behaviors and vulnerabilities that increase exposure to contaminated environmental media such as higher prevalence of outdoor work (M. Li et al., 2023, 2025; Usmani et al., 2023). As described in Section 3.2, this analysis conservatively estimates the extent to which the southeastern U.S. may present an area of elevated concern for microbiological exposures because it characterizes pathogenicity in relatively pristine soils. We do not directly account for risks that may derive from flooding of agricultural facilities which are densely concentrated in the southeastern U.S. This adds to the risks directly characterized here.

### 3.5 Drinking Water System Vulnerability

On a national basis, there were no correlations between relative abundance of RG2 or RG3 and prevalence of well water usage. T‐tests for relative abundance of RG1, RG2, and RG3 in public water system (PWS) versus non‐PWS areas were all insignificant. X^2^ tests comparing number of PWS versus non‐PWS regions by number of samples above different thresholds (e.g., 75th, 90th percentile) were insignificant. Therefore, the spatial scale over which trends in the relative abundance of RG2 and RG3 are observed (regional) is greater than the spatial scale on which we observe variability in water supply source (local). That is, in regions of higher versus lower RG2 and RG3 do not differ significantly in the proportion of the population served by public versus private water.

Nevertheless, within regions of higher RG2 and RG3, we would expect (predominantly rural) well water users to be more vulnerable because (a) they lack the surveillance infrastructure of public systems that would alert users to contamination and issue boil‐water or do‐not‐drink advisories in the event of contamination; (b) groundwater resources may be more vulnerable to persistent contamination due to longer recharge times than surface waters; and (c) well water users are disproportionately rural and socially vulnerable, introducing susceptibilities described in Section 3.4.

## 4 Conclusions

Putative pathogens are broadly distributed across soils of the contiguous United States, with highest abundance of putative pathogens in risk groups RG2 and RG3 predominantly found in forested ecoregions in the eastern and southeastern U.S. A novel statistical model developed here (a) returned results broadly consistent with previous work, for example, uncovering the significant role of moisture, organic matter, and nutrients in driving abundance of putative bacterial pathogens (Green et al., 1974; Jiang et al., 2021; Lekberg et al., 2021; Zaret et al., 2023); (b) revealed that these natural variables have more explanatory power than anthropogenic, land‐use processes on a regional basis; and (c) highlighted the significant national variability in the putative pathogenicity of pristine soils. The declining role of anthropogenic (as compared to natural) variables and declining model performance with increased relative abundance of soil pathogens reveals the potential for broadly distributed pathogenic risks, even in environments without obvious anthropogenic sources.

Emerging research has identified several common attributes of pathogens suggesting that they may systematically favor certain environmental conditions, and that those same attributes may select for pathogenicity (Naor‐Hoffmann et al., 2022). While the present analysis measures only putative pathogenicity using enrichment in taxa linked to RG2 and RG3, it is consistent with previous work suggesting a mechanistic basis for the selection of pathogens in certain environments. The relatively higher abundance of RG3 putative pathogens in the eastern temperate forest and tropical wet forest ecoregions can be attributed to multiple ecological factors that favor their persistence and proliferation. These ecoregions are characterized by warm temperatures, high humidity, and stable climatic conditions that provide ideal environments for the survival of mesophilic RG3 pathogens such as *M. tuberculosis* and *Yersinia pestis* (Barbieri et al., 2020). Also, the soils in these biomes are typically rich in organic matter and plant‐derived carbon inputs, which sustain complex microbial communities that include pathogens (Tian et al., 2021). Furthermore, the eastern temperate and tropical wet forest biomes host high biodiversity, encompassing a wide range of mammals, birds, reptiles, and arthropods that can act as natural reservoirs or vectors of RG3 pathogens. For example, *M*. *tuberculosis* complex relatives, most notably *M*. *bovis*, are maintained in multiple wildlife hosts (e.g., European badgers, white‐tailed deer, wild boar, African buffalo, brushtail possums); in general, forested landscapes host diverse mammalian reservoirs of pathogenic bacteria (M. V. Palmer, 2013). *Burkholderia pseudomallei*, an environmental saprophyte of tropical soils and waters, has documented infections across domestic and wild animals, evidence that mammal and reptile communities in warm, wet biomes can harbor RG3 organisms (Dance, 2000).

Frequent interactions between wildlife, soil, and water systems increase opportunities for environmental seeding and pathogen maintenance in forest ecosystems. Soil pathogens are mobilized to human receptors by natural processes including fire, flood, and wind, which may become more frequent and/or increase in intensity with climate change. While broadly distributed hotspots of putative pathogens are observed across the U.S., including apparent hotspots in the Midwest and California, high abundance of putative pathogens intersects most markedly with elevated mobilization hazards in the southeastern U.S. This includes notably proximity to surface waters, which is both a predictor of relative abundance of putative pathogens (possibly due to nutrient/moisture effects), and a risk factor for mobilization. The southeastern U.S. is also home to rural, low‐income and/or otherwise socially vulnerable populations who face potentially greater risks from pathogen exposure due to high preexisting burden of disease, low access to care, high prevalence of outdoor work, and other factors.

This screening analysis of how relative abundance of putative pathogens intersects with (a) relative risk for fire‐, flood‐, and/or wind‐driven pathogen mobilization and (b) relative social vulnerability of human receptors is intended to identify priority areas for follow‐up study, not to estimate numerical exposure magnitude. In particular, screening for intersections of higher RG2 or RG3 abundance with higher composite wind × fire × flood risk implicitly treats those physical mobilization phenomena equally, which is a major simplification. In reality, these three hazards will combine to affect exposure in ways that require detailed site‐specific analysis. Alternative weighting schemes were considered for this work but were ultimately deemed to be of little extra value because of (a) the limited scientific basis to propose schemes other than the all‐equal one retained here and (b) the finding that, across individual hazards and social vulnerability scores, the eastern and/or southeastern U.S. emerged as a priority area and was likely to do so for any weighting scheme we retained.

The data analyzed here correspond to environments spanning a range of land use profiles and natural environments but, to our knowledge, do not reflect specific environmental contamination or capture post‐mobilizing events. For example, proximity to agricultural sources is a known risk factor for pathogen exposure following heavy rains, but this analysis did not find pasture/cropland to be a significant predictor of relative abundance of putative soil pathogens. A finer‐scale process‐based model is needed to understand how these risks compare by diffuse reservoirs of pathogens in relatively pristine soils as characterized here. As described earlier, the southeastern U.S. is also a highly agriculturally diverse setting and would be a strategic location for this type of analysis.

This investigation differs from previous studies by examining variability in putative pathogenicity across a large (i.e., continental) spatial scale. Strong spatial clustering and the moderate predictive power of a statistical model suggest a combination of land‐use and natural drivers of these differences, as discussed earlier. Supplemental sampling at an intermediate spatial scale (e.g., watershed) around a subset of sample locations would add more information on how the variability at the national scale compares to more “intrinsic” variability within an environment with similar land‐use and other physical characteristics. Similarly, while this analysis has identified the southeastern U.S. as a priority area for analysis (especially when mobilization hazards are combined), there are hotspots in other regions as well, and these intersect with individual mobilization hazards, for example, fire in the western U.S. More intensive sampling at different scales and at different locations is likely to reveal other priorities for measuring and forecasting interactions between pathogen abundance, climate‐linked mobilization hazard, and receptor vulnerability. Overall, this analysis has sought to identify priority areas for follow‐up work rather than conclusively ranking exposure risks, which vary according to many site‐specific phenomena not addressed here.

While this analysis suggests that the diverse bacterial communities contributing to risk groups RG2 and RG3 are collectively influenced by natural properties of soils (notably in comparison to land‐use drivers), further species‐specific analysis would help elucidate what environmental mechanisms (e.g., adaptation to oxic‐anoxic cycling, etc.) select for which species, where they are likely to occur, and where more targeted analysis and surveillance may be warranted. This would also help strengthen linkages with analysis of the impacts of a changing climate; most research in this space has found that climate change is likely to favor pathogenic bacteria overall (especially in the agricultural regions that have been the focus of that research), but little is known about how individual pathogens may be favored by specific environmental changes.

Finally, this analysis has not quantified the burden of disease attributable to natural reservoirs of putative soil pathogens in comparison to anthropogenic sources (e.g., sewage overflows and downstream transport). Further research is needed to understand to what extent the hazards identified here translate into human health impact; this may include mechanistic exposure modeling as described previously and analysis of human health (e.g., emergency department) data across categories defined according to levels of natural and anthropogenic microbiological hazard. As described above, a large and growing body of research clearly identifies the potential for climate‐linked pathogen‐mobilizing events to cause disease (e.g., floods leading to increased exposures to waterborne illness). Yet, overall, more research is needed to characterize the role of natural reservoirs of soil pathogens as compared to anthropogenic sources such as sewer overflows and agricultural sites.

## Conflict of Interest

The authors declare no conflicts of interest relevant to this study.

## Supporting information

Supporting Information S1

## Data Availability

Raw soil data (relative abundance of putative pathogens by risk group, soil properties, nutrient abundance) for 622 samples identified by latitude and longitude is reported by Liao, Guo, et al. (2021). Inventory of sites on the National Priority List (NPL) is available from U.S. EPA (2025a). Inventory of sites in the Risk Management Program (RMP) is available from U.S. EPA (2025b). Emissions data from the National Pollutant Discharge and Elimination System (NPDES) is available from U.S. EPA (2025c). Hydrography data (to calculate distance to waterbody) are available from the National Hydrography Data set (USGS, 2023). Meteorological data (maximum and minimum temperatures, mean wind speed, and mean precipitation) are available from NOAA (2025). A full list of all variables with references is also included in Table S2 in Supporting Information S1.
