# Supplementary material for: Hotspots of Bacterial Pathogen Abundance and Exposure Risk in Soils of the Contiguous United States
Source: Geohealth. 2025 Dec 11;9(12):e2025GH001459. doi: 10.1029/2025GH001459 (PMC12698510; doi:10.1029/2025GH001459)
Supplement: Supplementary file 1 — Supporting Information S1 [file GH2-9-e2025GH001459-s001.pdf]

Hotspots of bacterial pathogen abundance and exposure risk in the contiguous United States

Supplemental information

Emily A. Matthews[a], Ying-Xian Goh[a,b], Shannon L. Hepp[a], Jingqiu Liao[a,b,c*], Ryan S.D. Calder[c,d,e*]

[a] Department of Civil and Environmental Engineering, Virginia Tech, Blacksburg, VA, 24061
[b] Center for Emerging, Zoonotic, and Arthropod-Borne Pathogens, Virginia Tech, Blacksburg, VA, 24061
[c] Global Change Center, Virginia Tech, Blacksburg, VA, 24061
[d] Department of Population Health Sciences, Virginia Tech, Blacksburg, VA, 24061
[e] Department of Civil and Environmental Engineering, Duke University, Durham, NC, 27708

* Corresponding authors' emails: liaoj@vt.edu, rsdc@vt.edu

List of tables

Table S1: List of pathogens included in each risk group (RG) 2
Table S2: Descriptions and references for predictor variables 3
Table S3: Random forest variable importance (main model) – RG1 8
Table S4: Random forest variable importance (main model) – RG2 9
Table S5: Random forest variable importance (main model) – RG3 10
Table S6: Random forest variable importance (spatial variables added) – RG1 11
Table S7: Random forest variable importance (spatial variables added) – RG2 12
Table S8: Random forest variable importance (spatial variables added) – RG3 13
Table S9: Random forest variable importance (collinear variables dropped) – RG1 14
Table S10: Random forest variable importance (collinear variables dropped) – RG2 15
Table S11: Random forest variable importance (collinear variables dropped) – RG3 16
Table S12: Random forest variable importance (in-group collinear variables dropped) – RG1 .. 17
Table S13: Random forest variable importance (in-group collinear variables dropped) – RG2 .. 18
Table S14: Random forest variable importance (in-group collinear variables dropped) – RG3 .. 19

List of figures

Figure S1: Distribution of U.S. Census Block Groups (bars) and pathogen sampling stations (points) by Risk Factor Score. 4
Figure S2: Frequency, correlation, and distribution of putative pathogens across the contiguous United States 5
Figure S3: Relative abundance of putative pathogens by state 6
Figure S4: Relative abundance of putative pathogens by ecoregion 7
Figure S5: First Street Foundation Risk Score maps for fire (a), flood (b), and wind (c). 20
Figure S6: Intersection of relative abundance of RG2 and RG3 with First Street Foundation Risk Score maps for fire, flood and wind 21
Figure S7: Intersection of relative abundance of RG2 and RG3 by Social Vulnerability Index percentile by county by thematic area 22

References 23

Table S1: List of pathogens included in each risk group (RG)

RG1	RG2	RG3
Bacillus circulans, Bacillus licheniformis, Bacillus pumilus, Bacillus subtilis, Lactococcus lactis, Microbacterium oxydans, Micrococcus luteus, Paenibacillus alvei, Sphingomonas melonis.	Achromobacter xylosoxidans, Acinetobacter baumannii, Acinetobacter calcoaceticus, Acinetobacter junii, Acinetobacter lwoffii, Acinetobacter radioresistens, Aeromonas hydrophila. Bacillus cereus, Bacillus thuringiensis, Burkholderia cepacian, Cedecea davisae, Citrobacter freundii. Clostridium perfringens, Coxiella burnetiid, Enterococcus faecalis, Escherichia coli Hafnia alvei, Klebsiella pneumoniae, Mycobacterium avium, Ochrobactrum anthropi, Pantoea agglomerans, Pseudomonas fluorescens, Pseudomonas putida, Pseudomonas stutzeri, Serratia marcescens, Staphylococcus saprophyticus, Stenotrophomonas maltophilia, Yersinia frederiksenii	Bartonella elizabethae, Brucella melitensis, Mycobacterium tuberculosis

Table S2: Descriptions and references for predictor variables

Predictor variable and description	Reference[a]
Natural properties	
Aluminum (Al) concentration (mg/kg)	
Average daily maximum temperature (ºC) 1985–2011	NOAA (2025)
Average daily minimum temperature (ºC) 1985–2011	NOAA (2025)
Calcium (Ca) concentration (mg/kg)	
Carbon (C) concentration (mg/kg)	
Copper (Cu) concentration (mg/kg)	
Distance to stream (m) – linear distance to closest vertex of a waterbody in National Hydrography Dataset	USGS (2023)
Iron (Fe) concentration (mg/kg)	
Magnesium (Mg) concentration (mg/kg)	
Manganese (Mn) concentration (mg/kg)	
Mean daily precipitation (mm)	NOAA (2025)
Mean daily wind speed (m/s)	NOAA (2025)
Molybdenum (Mb) concentration (mg/kg)	
Nitrogen (N) concentration (mg/kg)	
Organic matter (mg/kg)	
Potassium (K) concentration (mg/kg)	
pH	
Phosphorus (P) concentration (mg/kg)	
Sodium (Na) concentration (mg/kg)	
Soil moisture (%) – volumetric water content	
Sulfur (S) concentration (mg/kg)	
Zinc (Zn) concentration (mg/kg)	
Landscape properties	
Barren landscape (% within 10 km)	
Cropland (% within 10 km)	
Development ≥ 20% of area (% within 10 km)	
Development < 20% of area (% within 10 km)	
Forest (% within 10 km)	
Grassland (% within 10 km)	
National Pollutant Discharge and Elimination System (sites within 10 km)	U.S. EPA (2025a)
National Priority List (sites within 10 km)	U.S. EPA (2025b)
Open water (% within 10 km)	
Pasture (% within 10 km)	
Risk Management Program (sites within 10 km)	U.S. EPA (2025c)
Shrubland (% within 10 km)	
Treatment, Storage, and Disposal Facilities (sites within 10 km)	U.S. EPA (2025c)
Wetland (% within 10 km)	
Residual spatial features	
Longitude	
Latitude	
Elevation	

[a]Variables are from Liao et al. (2021) unless otherwise noted

Figure S1: Distribution of U.S. Census Block Groups (bars) and pathogen sampling stations (points) by Risk Factor Score. Score is calculated as weighted average of properties in each Risk Factor category for each hazard as described in the main text.

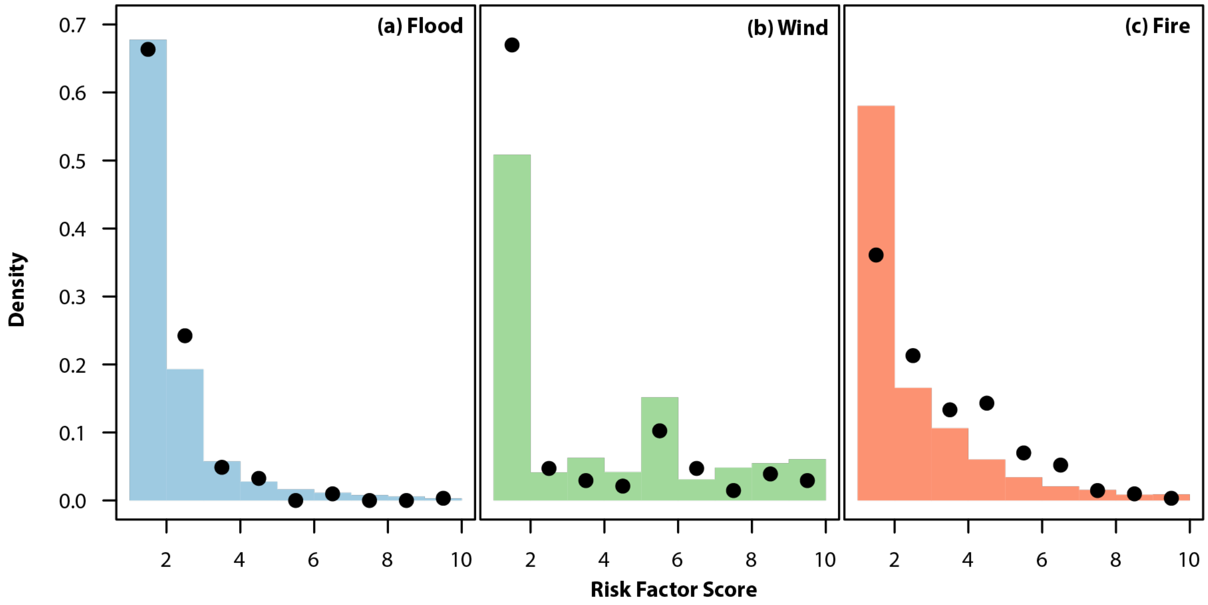

Figure S2: Frequency, correlation, and distribution of putative pathogens across the contiguous United States. (a) Mean relative abundance of all risk groups (RGs) pooled together, RG1, RG2, and RG3 putative pathogens; (b) Correlation between diversity as measured by Shannon-Wiener diversity index and the relative abundance of all pathogens; Relative abundance of pathogens across RG1 (c), RG2 (d), RG3 (e), and all risk groups combined (f); and (g) Diversity of pathogens as measured by Shannon diversity index across the contiguous United States. Projection: Albers CONUS.

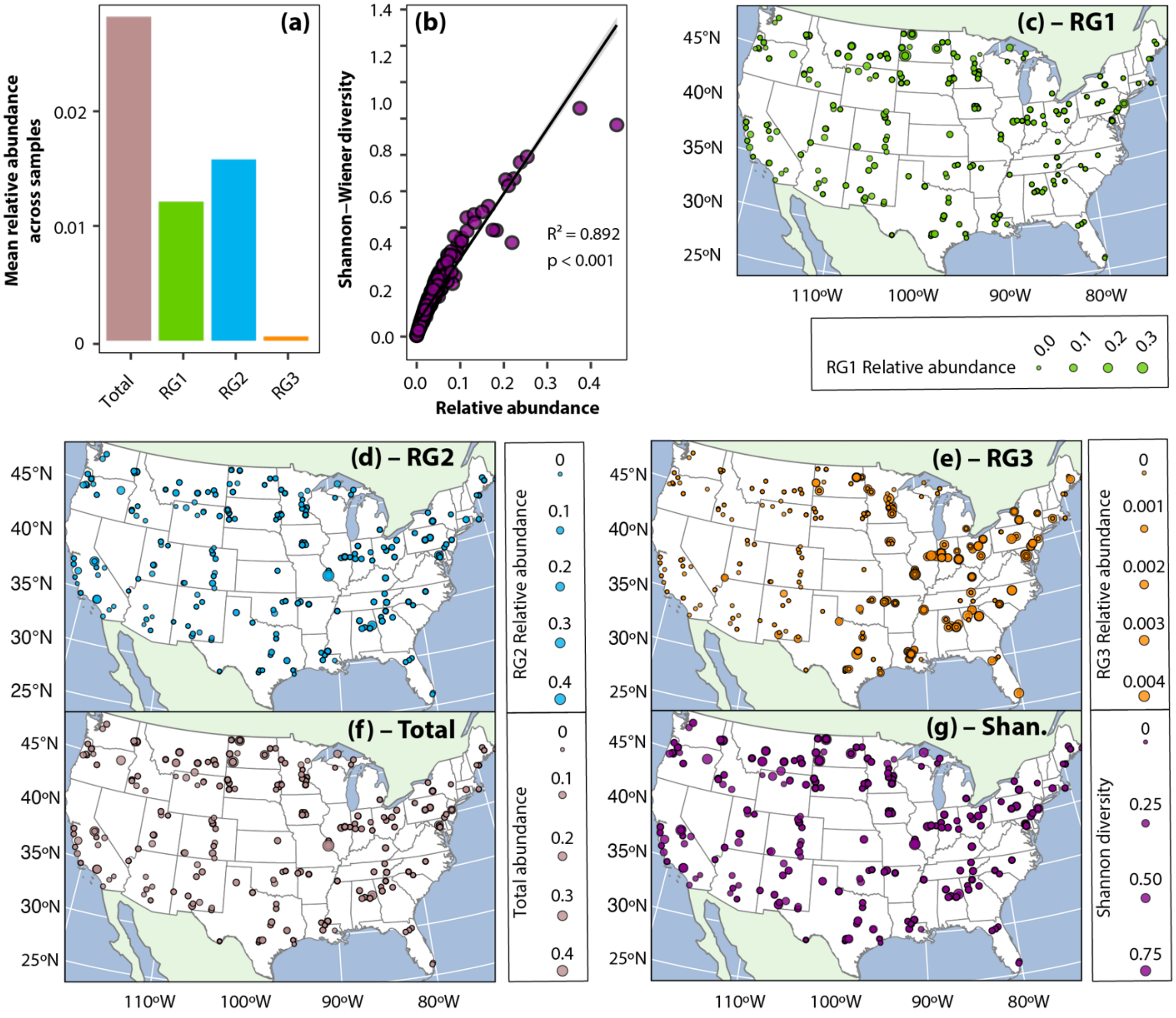

Figure S3: Relative abundance of putative pathogens by state for risk group (RG) 1 (a), RG2 (b), and RG3 (c).

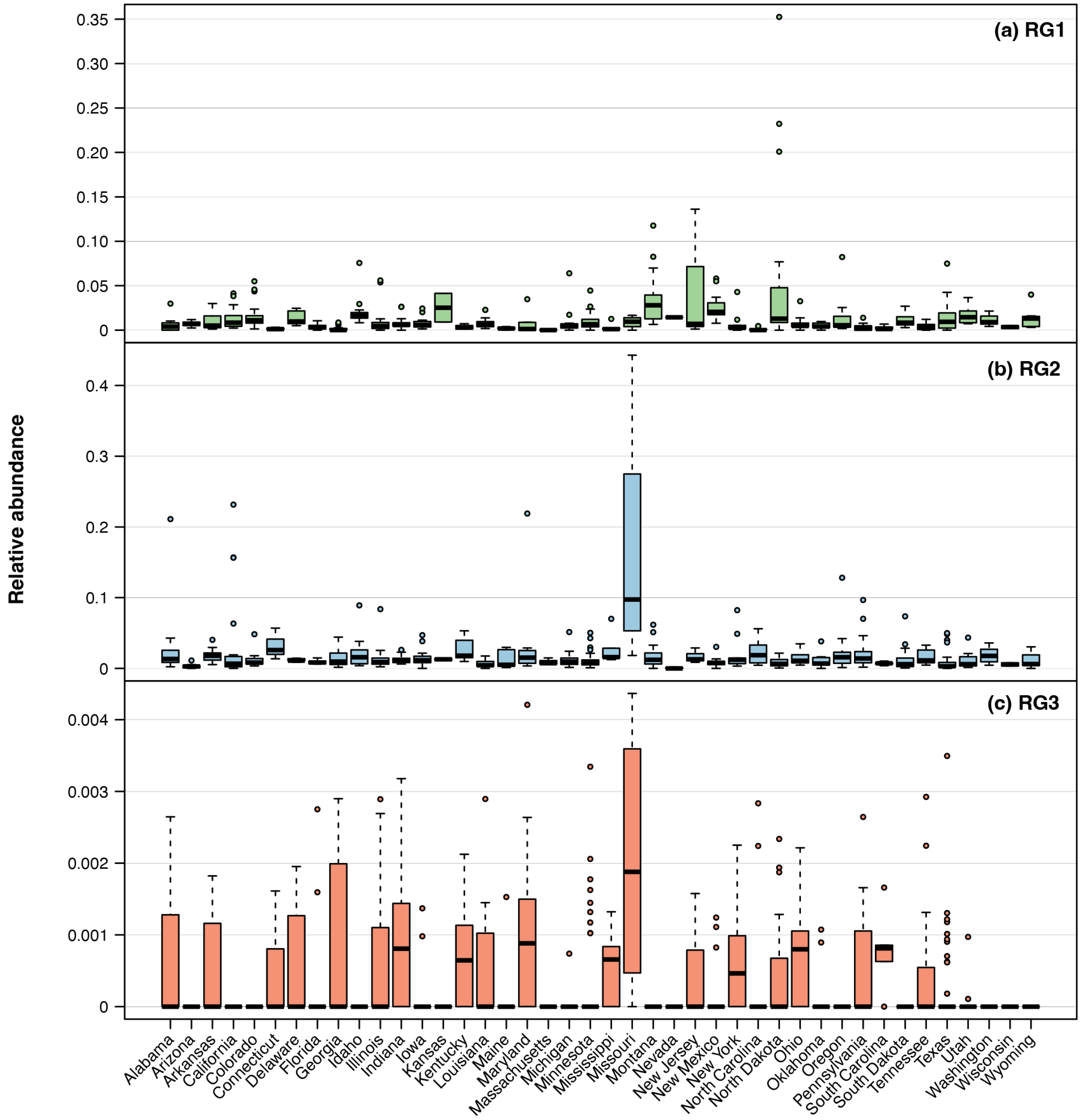

Figure S4: Relative abundance of putative pathogens by ecoregion for risk group (RG) 1 (a), RG2 (b), and RG3 (c).

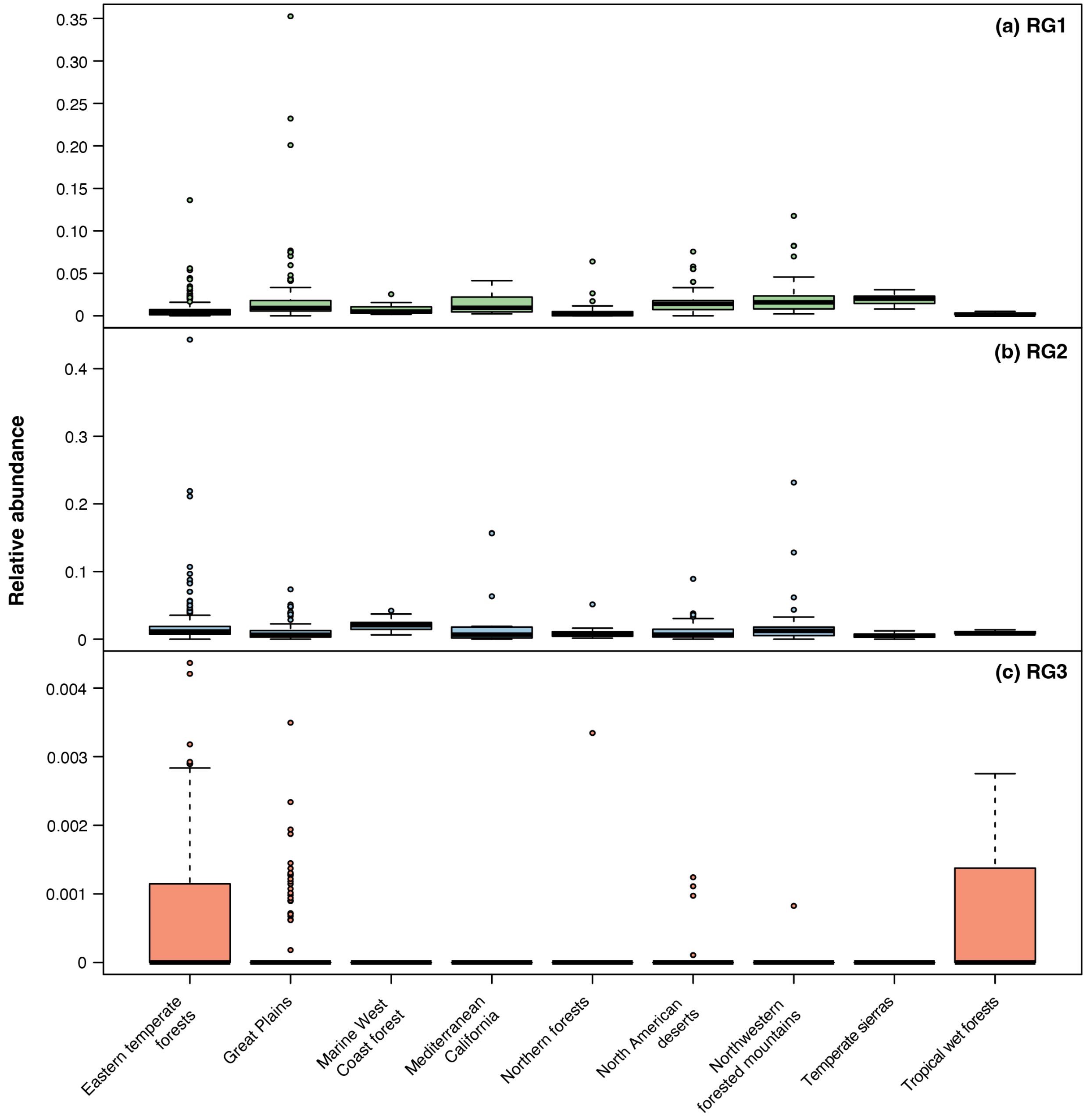

Table S3: Random forest variable importance (main model) – RG1 (18% of var explained)

Variable	Importance	Category
Mean daily precipitation (mm)	6.46E-03	Natural
Shrubland (% within 10 km)	4.82E-03	Landscape
Grassland (% within 10 km)	3.45E-03	Landscape
Open water (% within 10 km)	3.14E-03	Landscape
Molybdenum (Mb) concentration (mg/kg)	2.84E-03	Natural
Iron (Fe) concentration (mg/kg)	2.70E-03	Natural
Soil moisture (%) – volumetric water content	2.63E-03	Natural
Sodium (Na) concentration (mg/kg)	2.61E-03	Natural
Distance to stream (m) – linear distance to closest vertex of a waterbody in National Hydrography Dataset	2.56E-03	Natural
Development ≥ 20% of area (% within 10 km)	2.52E-03	Landscape
Development < 20% of area (% within 10 km)	2.48E-03	Landscape
Pasture (% within 10 km)	2.43E-03	Landscape
Cropland (% within 10 km)	2.22E-03	Landscape
Zinc (Zn) concentration (mg/kg)	2.14E-03	Natural
Wetland (% within 10 km)	1.86E-03	Landscape
Forest (% within 10 km)	1.75E-03	Landscape
Average daily minimum temperature (ºC) 1985–2011	1.56E-03	Natural
Potassium (K) concentration (mg/kg)	1.53E-03	Natural
Calcium (Ca) concentration (mg/kg)	1.49E-03	Natural
Manganese (Mn) concentration (mg/kg)	1.49E-03	Natural
Magnesium (Mg) concentration (mg/kg)	1.49E-03	Natural
Phosphorus (P) concentration (mg/kg)	1.43E-03	Natural
Barren landscape (% within 10 km)	1.42E-03	Landscape
pH	1.41E-03	Natural
Average daily maximum temperature (ºC) 1985–2011	1.37E-03	Natural
Carbon (C) concentration (mg/kg)	1.35E-03	Natural
Organic matter concentration (mg/kg)	1.34E-03	Natural
Aluminum (Al) concentration (mg/kg)	1.30E-03	Natural
Nitrogen (N) concentration (mg/kg)	1.17E-03	Natural
Copper (Cu) concentration (mg/kg)	1.17E-03	Natural
Sulfur (S) concentration (mg/kg)	1.06E-03	Natural
Mean daily wind speed (m/s)	9.42E-04	Natural
National Priority List (sites within 10 km)	6.66E-04	Landscape
Risk Management Program (sites within 10 km)	3.68E-04	Landscape
Treatment, Storage, and Disposal Facilities (sites within 10 km)	1.05E-04	Landscape
National Pollutant Discharge and Elimination System (sites within 10 km)	8.29E-05	Landscape

Table S4: Random forest variable importance (main model) – RG2 (14% of var explained)

Variable	Importance	Category
Open water (% within 10 km)	5.20E-03	Landscape
Iron (Fe) concentration (mg/kg)	5.07E-03	Natural
Nitrogen (N) concentration (mg/kg)	4.99E-03	Natural
Cropland (% within 10 km)	4.71E-03	Landscape
Carbon (C) concentration (mg/kg)	3.28E-03	Natural
Phosphorus (P) concentration (mg/kg)	2.79E-03	Natural
Pasture (% within 10 km)	2.74E-03	Landscape
Sodium (Na) concentration (mg/kg)	2.68E-03	Natural
Forest (% within 10 km)	2.67E-03	Landscape
Barren landscape (% within 10 km)	2.55E-03	Landscape
Grassland (% within 10 km)	2.48E-03	Landscape
Organic matter concentration (mg/kg)	2.42E-03	Natural
Zinc (Zn) concentration (mg/kg)	2.37E-03	Natural
Aluminum (Al) concentration (mg/kg)	2.33E-03	Natural
Manganese (Mn) concentration (mg/kg)	2.31E-03	Natural
Distance to stream (m) – linear distance to closest vertex of a waterbody in National Hydrography Dataset	2.31E-03	Natural
Shrubland (% within 10 km)	2.21E-03	Landscape
Copper (Cu) concentration (mg/kg)	2.17E-03	Natural
pH	2.17E-03	Natural
Calcium (Ca) concentration (mg/kg)	2.16E-03	Natural
Molybdenum (Mb) concentration (mg/kg)	2.12E-03	Natural
Wetland (% within 10 km)	2.07E-03	Landscape
Potassium (K) concentration (mg/kg)	2.03E-03	Natural
Mean daily precipitation (mm)	2.01E-03	Natural
Magnesium (Mg) concentration (mg/kg)	1.82E-03	Natural
Soil moisture (%) – volumetric water content	1.73E-03	Natural
Development ≥ 20% of area (% within 10 km)	1.73E-03	Landscape
Mean daily wind speed (m/s)	1.68E-03	Natural
Average daily minimum temperature (ºC) 1985–2011	1.67E-03	Natural
Sulfur (S) concentration (mg/kg)	1.62E-03	Natural
Average daily maximum temperature (ºC) 1985–2011	1.56E-03	Natural
Development < 20% of area (% within 10 km)	1.49E-03	Landscape
National Pollutant Discharge and Elimination System (sites within 10 km)	9.31E-04	Landscape
National Priority List (sites within 10 km)	7.26E-04	Landscape
Risk Management Program (sites within 10 km)	4.59E-04	Landscape
Treatment, Storage, and Disposal Facilities (sites within 10 km)	4.61E-05	Landscape

Table S5: Random forest variable importance (main model) – RG3 (6% of var explained)

Variable	Importance	Category
Manganese (Mn) concentration (mg/kg)	3.99E-06	Natural
Zinc (Zn) concentration (mg/kg)	2.56E-06	Natural
Phosphorus (P) concentration (mg/kg)	2.01E-06	Natural
Mean daily wind speed (m/s)	1.98E-06	Natural
Distance to stream (m) – linear distance to closest vertex of a waterbody in National Hydrography Dataset	1.55E-06	Natural
National Pollutant Discharge and Elimination System (sites within 10 km)	1.54E-06	Landscape
Mean daily precipitation (mm)	1.52E-06	Natural
Carbon (C) concentration (mg/kg)	1.51E-06	Natural
Aluminum (Al) concentration (mg/kg)	1.36E-06	Natural
pH	1.20E-06	Natural
Average daily maximum temperature (ºC) 1985–2011	1.18E-06	Natural
Calcium (Ca) concentration (mg/kg)	1.18E-06	Natural
Potassium (K) concentration (mg/kg)	1.18E-06	Natural
Sulfur (S) concentration (mg/kg)	1.13E-06	Natural
Organic matter concentration (mg/kg)	1.12E-06	Natural
Soil moisture (%) – volumetric water content	1.09E-06	Natural
Nitrogen (N) concentration (mg/kg)	1.08E-06	Natural
Barren landscape (% within 10 km)	1.06E-06	Landscape
Copper (Cu) concentration (mg/kg)	1.04E-06	Natural
Open water (% within 10 km)	1.02E-06	Landscape
Grassland (% within 10 km)	1.01E-06	Landscape
Cropland (% within 10 km)	9.60E-07	Landscape
Sodium (Na) concentration (mg/kg)	9.06E-07	Natural
Iron (Fe) concentration (mg/kg)	8.90E-07	Natural
Magnesium (Mg) concentration (mg/kg)	8.69E-07	Natural
Shrubland (% within 10 km)	8.42E-07	Landscape
Forest (% within 10 km)	8.17E-07	Landscape
Development < 20% of area (% within 10 km)	7.98E-07	Landscape
Development ≥ 20% of area (% within 10 km)	7.79E-07	Landscape
Average daily minimum temperature (ºC) 1985–2011	7.60E-07	Natural
Molybdenum (Mb) concentration (mg/kg)	7.59E-07	Natural
Wetland (% within 10 km)	7.59E-07	Landscape
Pasture (% within 10 km)	6.82E-07	Landscape
Risk Management Program (sites within 10 km)	2.77E-07	Landscape
Treatment, Storage, and Disposal Facilities (sites within 10 km)	9.85E-08	Landscape
National Priority List (sites within 10 km)	7.46E-08	Landscape

Table S6: Random forest variable importance (spatial variables added) – RG1 (20% of var explained)

Variable	Importance	Category
Longitude	4.35E-03	Spatial
Shrubland (% within 10 km)	4.15E-03	Landscape
Mean daily precipitation (mm)	4.08E-03	Natural
Latitude	3.91E-03	Spatial
Elevation (m)	3.39E-03	Spatial
Sodium (Na) concentration (mg/kg)	2.76E-03	Natural
Iron (Fe) concentration (mg/kg)	2.60E-03	Natural
Molybdenum (Mb) concentration (mg/kg)	2.52E-03	Natural
Pasture (% within 10 km)	2.40E-03	Landscape
Development ≥ 20% of area (% within 10 km)	2.07E-03	Landscape
Grassland (% within 10 km)	1.93E-03	Landscape
Cropland (% within 10 km)	1.87E-03	Landscape
Development < 20% of area (% within 10 km)	1.83E-03	Landscape
Forest (% within 10 km)	1.75E-03	Landscape
Wetland (% within 10 km)	1.67E-03	Landscape
Open water (% within 10 km)	1.67E-03	Landscape
Soil moisture (%) – volumetric water content	1.60E-03	Natural
Sulfur (S) concentration (mg/kg)	1.56E-03	Natural
Calcium (Ca) concentration (mg/kg)	1.56E-03	Natural
Distance to stream (m) – linear distance to closest vertex of a waterbody in National Hydrography Dataset	1.50E-03	Natural
Zinc (Zn) concentration (mg/kg)	1.44E-03	Natural
Manganese (Mn) concentration (mg/kg)	1.36E-03	Natural
Aluminum (Al) concentration (mg/kg)	1.24E-03	Natural
Potassium (K) concentration (mg/kg)	1.24E-03	Natural
Phosphorus (P) concentration (mg/kg)	1.23E-03	Natural
Magnesium (Mg) concentration (mg/kg)	1.18E-03	Natural
Barren landscape (% within 10 km)	1.13E-03	Landscape
pH	1.13E-03	Natural
Average daily minimum temperature (ºC) 1985–2011	1.08E-03	Natural
Nitrogen (N) concentration (mg/kg)	1.07E-03	Natural
Average daily maximum temperature (ºC) 1985–2011	9.28E-04	Natural
Carbon (C) concentration (mg/kg)	9.16E-04	Natural
State	8.95E-04	Spatial
Organic matter concentration (mg/kg)	7.90E-04	Natural
Copper (Cu) concentration (mg/kg)	7.34E-04	Natural
Mean daily wind speed (m/s)	6.15E-04	Natural
Ecoregion	3.95E-04	Spatial
National Priority List (sites within 10 km)	3.71E-04	Landscape
Risk Management Program (sites within 10 km)	2.96E-04	Landscape

Table S7: Random forest variable importance (spatial variables added) – RG2 (12% of var explained)

Variable	Importance	Category
Nitrogen (N) concentration (mg/kg)	4.87E-03	Natural
Iron (Fe) concentration (mg/kg)	4.64E-03	Natural
Open water (% within 10 km)	3.96E-03	Landscape
Forest (% within 10 km)	3.88E-03	Landscape
Cropland (% within 10 km)	3.75E-03	Landscape
Latitude	3.35E-03	Spatial
Carbon (C) concentration (mg/kg)	3.35E-03	Natural
Calcium (Ca) concentration (mg/kg)	3.05E-03	Natural
pH	2.92E-03	Natural
Grassland (% within 10 km)	2.83E-03	Landscape
Sodium (Na) concentration (mg/kg)	2.63E-03	Natural
Molybdenum (Mb) concentration (mg/kg)	2.62E-03	Natural
Organic matter concentration (mg/kg)	2.53E-03	Natural
Longitude	2.47E-03	Spatial
Pasture (% within 10 km)	2.47E-03	Landscape
Distance to stream (m) – linear distance to closest vertex of a waterbody in National Hydrography Dataset	2.34E-03	Natural
Zinc (Zn) concentration (mg/kg)	2.33E-03	Natural
Barren landscape (% within 10 km)	2.20E-03	Landscape
Aluminum (Al) concentration (mg/kg)	2.18E-03	Natural
Copper (Cu) concentration (mg/kg)	2.15E-03	Natural
Manganese (Mn) concentration (mg/kg)	2.14E-03	Natural
Phosphorus (P) concentration (mg/kg)	2.10E-03	Natural
Potassium (K) concentration (mg/kg)	1.96E-03	Natural
Mean daily precipitation (mm)	1.95E-03	Natural
Shrubland (% within 10 km)	1.89E-03	Landscape
Wetland (% within 10 km)	1.85E-03	Landscape
Elevation (m)	1.84E-03	Spatial
Soil moisture (%) – volumetric water content	1.75E-03	Natural
Magnesium (Mg) concentration (mg/kg)	1.73E-03	Natural
Average daily minimum temperature (ºC) 1985–2011	1.65E-03	Natural
Development ≥ 20% of area (% within 10 km)	1.53E-03	Landscape
Sulfur (S) concentration (mg/kg)	1.43E-03	Natural
Development < 20% of area (% within 10 km)	1.30E-03	Landscape
Mean daily wind speed (m/s)	1.28E-03	Natural
Average daily maximum temperature (ºC) 1985–2011	1.19E-03	Natural
State	8.62E-04	Spatial
National Pollutant Discharge and Elimination System (sites within 10 km)	7.87E-04	Landscape
National Priority List (sites within 10 km)	7.72E-04	Landscape
Ecoregion	4.54E-04	Spatial

Table S8: Random forest variable importance (spatial variables added) – RG3 (3% of var explained)

Variable	Importance	Category
Manganese (Mn) concentration (mg/kg)	3.81E-06	Natural
Zinc (Zn) concentration (mg/kg)	2.28E-06	Natural
Mean daily wind speed (m/s)	1.53E-06	Natural
Phosphorus (P) concentration (mg/kg)	1.47E-06	Natural
Distance to stream (m) – linear distance to closest vertex of a waterbody in National Hydrography Dataset	1.44E-06	Natural
Carbon (C) concentration (mg/kg)	1.37E-06	Natural
National Pollutant Discharge and Elimination System (sites within 10 km)	1.36E-06	Landscape
Mean daily precipitation (mm)	1.34E-06	Natural
Sulfur (S) concentration (mg/kg)	1.31E-06	Natural
Longitude	1.26E-06	Spatial
State	1.19E-06	Spatial
Aluminum (Al) concentration (mg/kg)	1.14E-06	Natural
Calcium (Ca) concentration (mg/kg)	1.10E-06	Natural
Organic matter concentration (mg/kg)	1.05E-06	Natural
Copper (Cu) concentration (mg/kg)	1.02E-06	Natural
pH	1.02E-06	Natural
Potassium (K) concentration (mg/kg)	1.00E-06	Natural
Open water (% within 10 km)	9.56E-07	Landscape
Average daily maximum temperature (ºC) 1985–2011	9.50E-07	Natural
Barren landscape (% within 10 km)	9.35E-07	Landscape
Wetland (% within 10 km)	8.98E-07	Landscape
Soil moisture (%) – volumetric water content	8.62E-07	Natural
Sodium (Na) concentration (mg/kg)	8.40E-07	Natural
Nitrogen (N) concentration (mg/kg)	8.31E-07	Natural
Forest (% within 10 km)	8.28E-07	Landscape
Grassland (% within 10 km)	8.23E-07	Landscape
Elevation (m)	8.01E-07	Spatial
Magnesium (Mg) concentration (mg/kg)	7.85E-07	Natural
Pasture (% within 10 km)	7.72E-07	Landscape
Latitude	7.50E-07	Spatial
Development < 20% of area (% within 10 km)	7.19E-07	Landscape
Iron (Fe) concentration (mg/kg)	7.10E-07	Natural
Shrubland (% within 10 km)	7.06E-07	Landscape
Cropland (% within 10 km)	7.04E-07	Landscape
Molybdenum (Mb) concentration (mg/kg)	6.86E-07	Natural
Development ≥ 20% of area (% within 10 km)	6.69E-07	Landscape
Average daily minimum temperature (ºC) 1985–2011	6.50E-07	Natural
Risk Management Program (sites within 10 km)	1.30E-07	Landscape
Treatment, Storage, and Disposal Facilities (sites within 10 km)	1.13E-07	Landscape

Table S9: Random forest variable importance (collinear variables dropped) – RG1 (16% of var explained)

Variable	Importance	Category
Shrubland (% within 10 km)	7.44E-03	Landscape
Grassland (% within 10 km)	4.57E-03	Landscape
Iron (Fe) concentration (mg/kg)	4.05E-03	Natural
Soil moisture (%) – volumetric water content	3.99E-03	Natural
Cropland (% within 10 km)	3.95E-03	Landscape
Molybdenum (Mb) concentration (mg/kg)	3.80E-03	Natural
Zinc (Zn) concentration (mg/kg)	3.47E-03	Natural
Pasture (% within 10 km)	3.11E-03	Landscape
Forest (% within 10 km)	3.00E-03	Landscape
Sodium (Na) concentration (mg/kg)	2.85E-03	Natural
Organic matter concentration (mg/kg)	2.65E-03	Natural
Potassium (K) concentration (mg/kg)	2.54E-03	Natural
Calcium (Ca) concentration (mg/kg)	2.40E-03	Natural
Manganese (Mn) concentration (mg/kg)	2.40E-03	Natural
Barren landscape (% within 10 km)	2.36E-03	Landscape
Wetland (% within 10 km)	2.36E-03	Landscape
pH	2.31E-03	Natural
Aluminum (Al) concentration (mg/kg)	2.23E-03	Natural
Magnesium (Mg) concentration (mg/kg)	2.09E-03	Natural
Sulfur (S) concentration (mg/kg)	2.05E-03	Natural
Copper (Cu) concentration (mg/kg)	1.67E-03	Natural
Phosphorus (P) concentration (mg/kg)	1.62E-03	Natural
National Priority List (sites within 10 km)	7.50E-04	Landscape
Risk Management Program (sites within 10 km)	4.08E-04	Landscape
National Pollutant Discharge and Elimination System (sites within 10 km)	2.41E-04	Landscape
Treatment, Storage, and Disposal Facilities (sites within 10 km)	1.08E-04	Landscape

Table S10: Random forest variable importance (collinear variables dropped) – RG2 (10% of var explained)

Variable	Importance	Category
Cropland (% within 10 km)	5.97E-03	Landscape
Iron (Fe) concentration (mg/kg)	5.75E-03	Natural
Organic matter concentration (mg/kg)	4.31E-03	Natural
Sodium (Na) concentration (mg/kg)	4.27E-03	Natural
Forest (% within 10 km)	4.14E-03	Landscape
Phosphorus (P) concentration (mg/kg)	4.07E-03	Natural
Shrubland (% within 10 km)	4.06E-03	Landscape
Barren landscape (% within 10 km)	4.04E-03	Landscape
Grassland (% within 10 km)	3.71E-03	Landscape
Zinc (Zn) concentration (mg/kg)	3.64E-03	Natural
Aluminum (Al) concentration (mg/kg)	3.60E-03	Natural
Pasture (% within 10 km)	3.33E-03	Landscape
Manganese (Mn) concentration (mg/kg)	3.32E-03	Natural
pH	3.28E-03	Natural
Copper (Cu) concentration (mg/kg)	3.04E-03	Natural
Potassium (K) concentration (mg/kg)	2.82E-03	Natural
Calcium (Ca) concentration (mg/kg)	2.80E-03	Natural
Molybdenum (Mb) concentration (mg/kg)	2.74E-03	Natural
Wetland (% within 10 km)	2.69E-03	Landscape
Soil moisture (%) – volumetric water content	2.65E-03	Natural
Magnesium (Mg) concentration (mg/kg)	2.50E-03	Natural
Sulfur (S) concentration (mg/kg)	2.23E-03	Natural
National Pollutant Discharge and Elimination System (sites within 10 km)	1.22E-03	Landscape
National Priority List (sites within 10 km)	1.10E-03	Landscape
Risk Management Program (sites within 10 km)	7.99E-04	Landscape
Treatment, Storage, and Disposal Facilities (sites within 10 km)	1.14E-04	Landscape

Table S11: Random forest variable importance (collinear variables dropped) – RG3 (4% of var explained)

Variable	Importance	Category
Manganese (Mn) concentration (mg/kg)	4.83E-06	Natural
Zinc (Zn) concentration (mg/kg)	3.16E-06	Natural
National Pollutant Discharge and Elimination System (sites within 10 km)	2.35E-06	Landscape
Phosphorus (P) concentration (mg/kg)	2.30E-06	Natural
Organic matter concentration (mg/kg)	1.92E-06	Natural
Aluminum (Al) concentration (mg/kg)	1.80E-06	Natural
Barren landscape (% within 10 km)	1.62E-06	Landscape
Sulfur (S) concentration (mg/kg)	1.61E-06	Natural
pH	1.59E-06	Natural
Soil moisture (%) – volumetric water content	1.59E-06	Natural
Potassium (K) concentration (mg/kg)	1.56E-06	Natural
Calcium (Ca) concentration (mg/kg)	1.54E-06	Natural
Forest (% within 10 km)	1.50E-06	Landscape
Grassland (% within 10 km)	1.45E-06	Landscape
Iron (Fe) concentration (mg/kg)	1.40E-06	Natural
Pasture (% within 10 km)	1.31E-06	Landscape
Copper (Cu) concentration (mg/kg)	1.31E-06	Natural
Molybdenum (Mb) concentration (mg/kg)	1.23E-06	Natural
Cropland (% within 10 km)	1.23E-06	Landscape
Magnesium (Mg) concentration (mg/kg)	1.23E-06	Natural
Wetland (% within 10 km)	1.19E-06	Landscape
Sodium (Na) concentration (mg/kg)	1.16E-06	Natural
Shrubland (% within 10 km)	1.01E-06	Landscape
Risk Management Program (sites within 10 km)	3.99E-07	Landscape
Treatment, Storage, and Disposal Facilities (sites within 10 km)	2.16E-07	Landscape
National Priority List (sites within 10 km)	1.16E-07	Landscape

Table S12: Random forest variable importance (in-group collinear variables dropped) – RG1 (16% of var explained)

Variable	Importance	Category
Shrubland (% within 10 km)	7.44E-03	Landscape
Grassland (% within 10 km)	4.57E-03	Landscape
Iron (Fe) concentration (mg/kg)	4.05E-03	Natural
Soil moisture (%) – volumetric water content	3.99E-03	Natural
Cropland (% within 10 km)	3.95E-03	Landscape
Molybdenum (Mb) concentration (mg/kg)	3.80E-03	Natural
Zinc (Zn) concentration (mg/kg)	3.47E-03	Natural
Pasture (% within 10 km)	3.11E-03	Landscape
Forest (% within 10 km)	3.00E-03	Landscape
Sodium (Na) concentration (mg/kg)	2.85E-03	Natural
Organic matter concentration (mg/kg)	2.65E-03	Natural
Potassium (K) concentration (mg/kg)	2.54E-03	Natural
Calcium (Ca) concentration (mg/kg)	2.40E-03	Natural
Manganese (Mn) concentration (mg/kg)	2.40E-03	Natural
Barren landscape (% within 10 km)	2.36E-03	Landscape
Wetland (% within 10 km)	2.36E-03	Landscape
pH	2.31E-03	Natural
Aluminum (Al) concentration (mg/kg)	2.23E-03	Natural
Magnesium (Mg) concentration (mg/kg)	2.09E-03	Natural
Sulfur (S) concentration (mg/kg)	2.05E-03	Natural
Copper (Cu) concentration (mg/kg)	1.67E-03	Natural
Phosphorus (P) concentration (mg/kg)	1.62E-03	Natural
National Priority List (sites within 10 km)	7.50E-04	Landscape
Risk Management Program (sites within 10 km)	4.08E-04	Landscape
National Pollutant Discharge and Elimination System (sites within 10 km)	2.41E-04	Landscape
Treatment, Storage, and Disposal Facilities (sites within 10 km)	1.08E-04	Landscape

Table S13: Random forest variable importance (in-group collinear variables dropped) – RG2 (10% of var explained)

Variable	Importance	Category
Cropland (% within 10 km)	5.97E-03	Landscape
Iron (Fe) concentration (mg/kg)	5.75E-03	Natural
Organic matter concentration (mg/kg)	4.31E-03	Natural
Sodium (Na) concentration (mg/kg)	4.27E-03	Natural
Forest (% within 10 km)	4.14E-03	Landscape
Phosphorus (P) concentration (mg/kg)	4.07E-03	Natural
Shrubland (% within 10 km)	4.06E-03	Landscape
Barren landscape (% within 10 km)	4.04E-03	Landscape
Grassland (% within 10 km)	3.71E-03	Landscape
Zinc (Zn) concentration (mg/kg)	3.64E-03	Natural
Aluminum (Al) concentration (mg/kg)	3.60E-03	Natural
Pasture (% within 10 km)	3.33E-03	Landscape
Manganese (Mn) concentration (mg/kg)	3.32E-03	Natural
pH	3.28E-03	Natural
Copper (Cu) concentration (mg/kg)	3.04E-03	Natural
Potassium (K) concentration (mg/kg)	2.82E-03	Natural
Calcium (Ca) concentration (mg/kg)	2.80E-03	Natural
Molybdenum (Mb) concentration (mg/kg)	2.74E-03	Natural
Wetland (% within 10 km)	2.69E-03	Landscape
Soil moisture (%) – volumetric water content	2.65E-03	Natural
Magnesium (Mg) concentration (mg/kg)	2.50E-03	Natural
Sulfur (S) concentration (mg/kg)	2.23E-03	Natural
National Pollutant Discharge and Elimination System (sites within 10 km)	1.22E-03	Landscape
National Priority List (sites within 10 km)	1.10E-03	Landscape
Risk Management Program (sites within 10 km)	7.99E-04	Landscape
Treatment, Storage, and Disposal Facilities (sites within 10 km)	1.14E-04	Landscape

Table S14: Random forest variable importance (in-group collinear variables dropped) – RG3 (4% of var explained)

Variable	Importance	Category
Manganese (Mn) concentration (mg/kg)	4.83E-06	Natural
Zinc (Zn) concentration (mg/kg)	3.16E-06	Natural
National Pollutant Discharge and Elimination System (sites within 10 km)	2.35E-06	Landscape
Phosphorus (P) concentration (mg/kg)	2.30E-06	Natural
Organic matter concentration (mg/kg)	1.92E-06	Natural
Aluminum (Al) concentration (mg/kg)	1.80E-06	Natural
Barren landscape (% within 10 km)	1.62E-06	Landscape
Sulfur (S) concentration (mg/kg)	1.61E-06	Natural
pH	1.59E-06	Natural
Soil moisture (%) – volumetric water content	1.59E-06	Natural
Potassium (K) concentration (mg/kg)	1.56E-06	Natural
Calcium (Ca) concentration (mg/kg)	1.54E-06	Natural
Forest (% within 10 km)	1.50E-06	Landscape
Grassland (% within 10 km)	1.45E-06	Landscape
Iron (Fe) concentration (mg/kg)	1.40E-06	Natural
Pasture (% within 10 km)	1.31E-06	Landscape
Copper (Cu) concentration (mg/kg)	1.31E-06	Natural
Molybdenum (Mb) concentration (mg/kg)	1.23E-06	Natural
Cropland (% within 10 km)	1.23E-06	Landscape
Magnesium (Mg) concentration (mg/kg)	1.23E-06	Natural
Wetland (% within 10 km)	1.19E-06	Landscape
Sodium (Na) concentration (mg/kg)	1.16E-06	Natural
Shrubland (% within 10 km)	1.01E-06	Landscape
Risk Management Program (sites within 10 km)	3.99E-07	Landscape
Treatment, Storage, and Disposal Facilities (sites within 10 km)	2.16E-07	Landscape
National Priority List (sites within 10 km)	1.16E-07	Landscape

Figure S5: First Street Foundation Risk Score maps for fire (a), flood (b), and wind (c). Score is calculated as weighted average of properties in each Risk Factor category for each hazard as described in the main text.

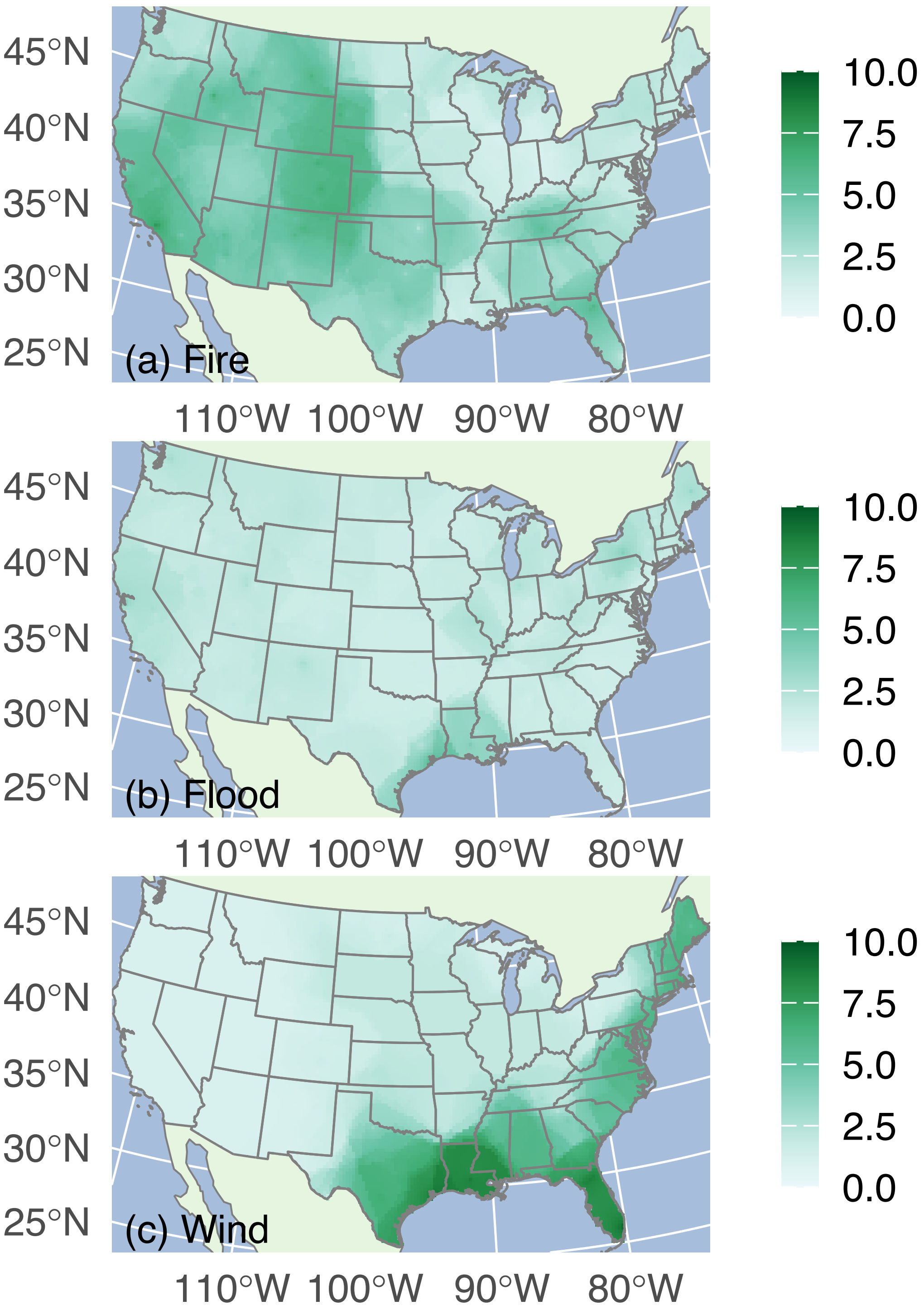

Figure S6: Intersection of relative abundance of RG2 and RG3 with First Street Foundation Risk Score maps for fire (RG2 and RG3 in a and b, respectively), flood (c and d), and wind (e and f). Score is calculated as weighted average of properties in each Risk Factor category for each hazard as described in the main text.

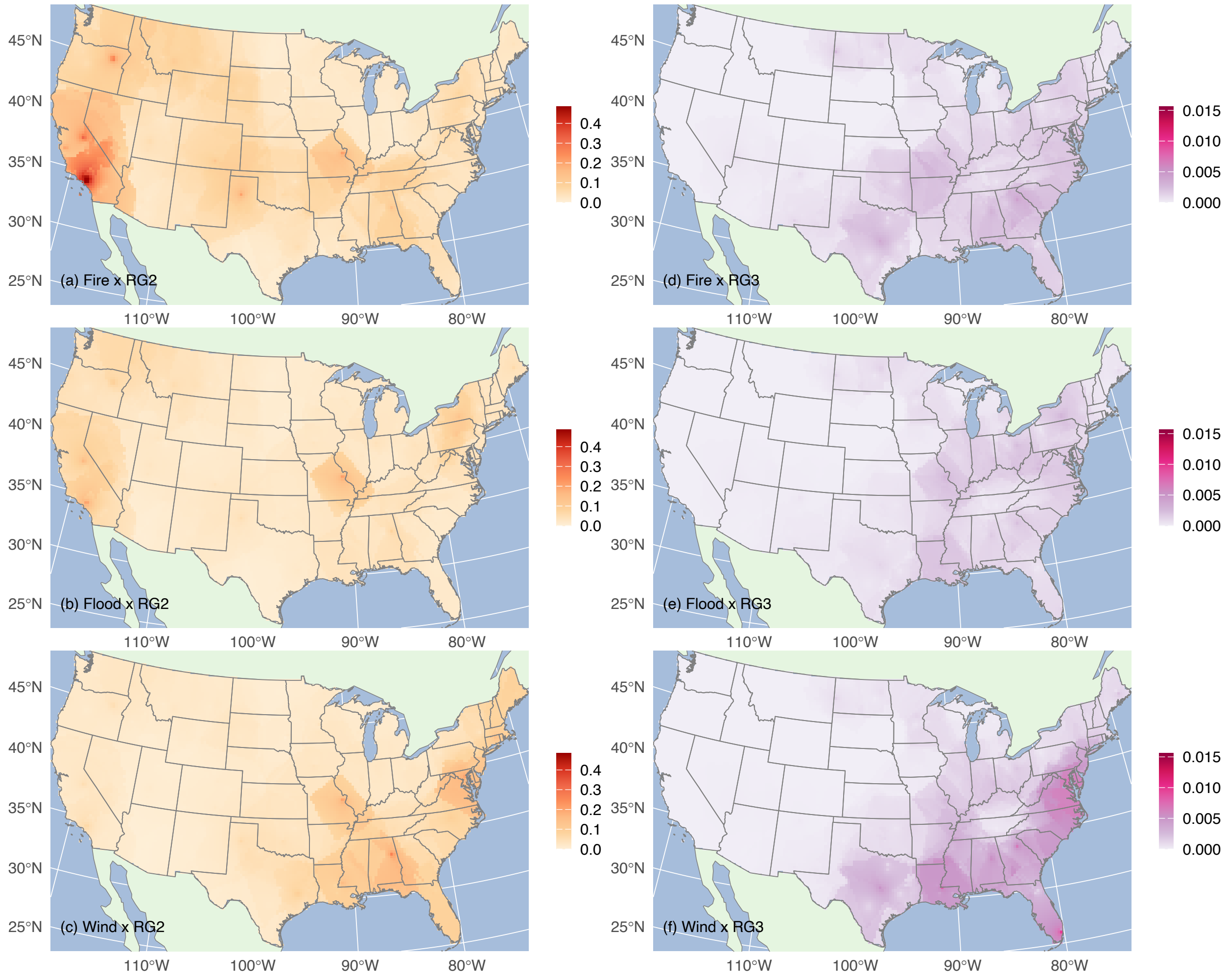

Figure S7: Intersection of relative abundance of RG2 and RG3 by Social Vulnerability Index percentile by county by thematic area: socioeconomic status (RG2 and RG3 in a and b, respectively); household characteristics (c and d); racial & ethnic minority status (e and f); and housing type & transportation (g and h).

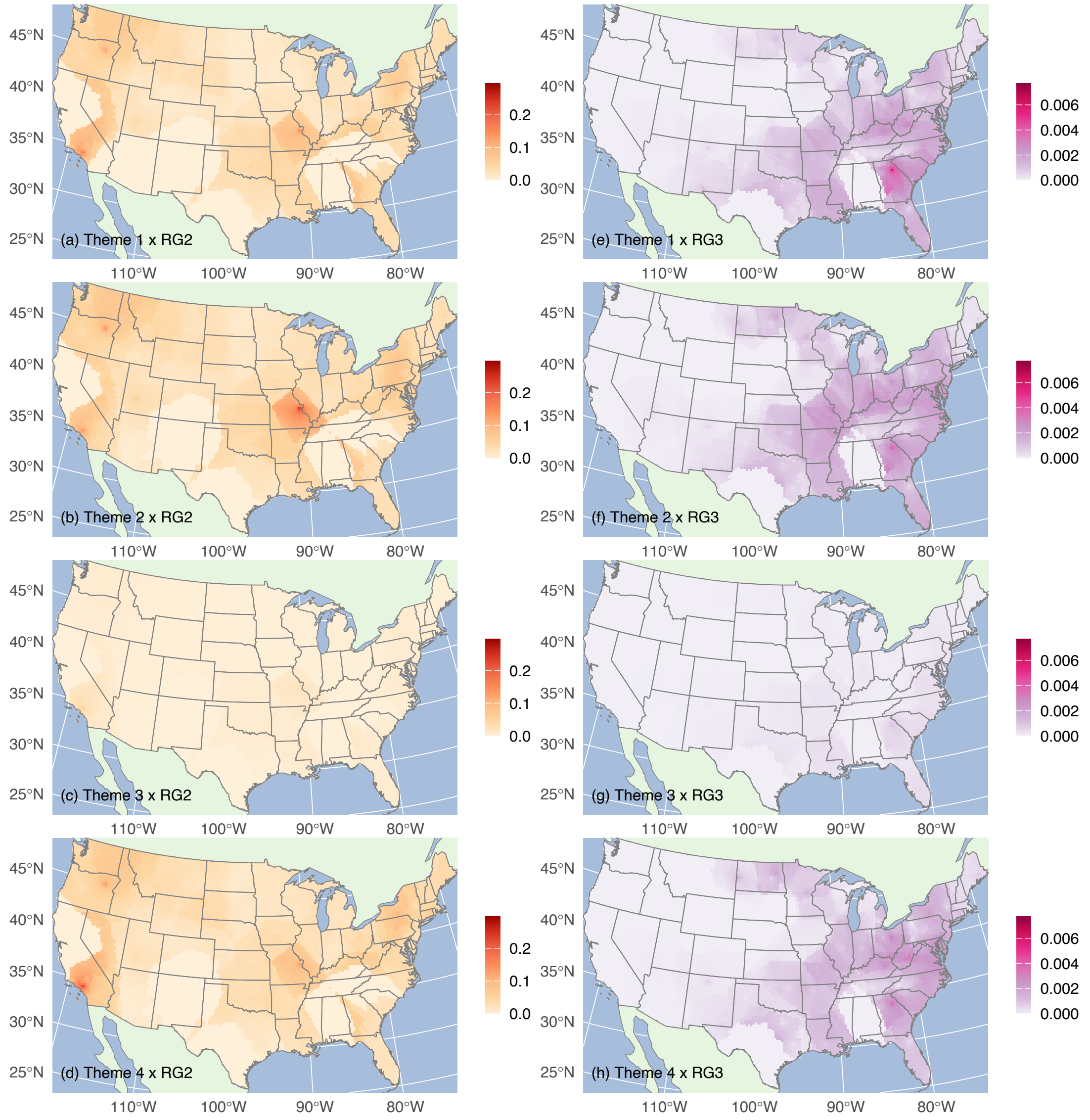

References

Liao, J., X. Guo, D. L. Weller, S. Pollak, D. H. Buckley, M. Wiedmann and O. X. Cordero (2021). "Nationwide genomic atlas of soil-dwelling Listeria reveals effects of selection and population ecology on pangenome evolution." Nature Microbiology **6**(8): 1021–1030.

National Oceanic and Atmospheric Administration (NOAA) Physical Sciences Laboratory. (2025). "Index of /Datasets/livneh/metvars." Dataset. Retrieved 2025-02-27 from https://downloads.psl.noaa.gov/Datasets/livneh/metvars/.

United States Environmental Protection Agency (U.S. EPA). (2025a). "NPL Superfund Site Boundaries." Dataset. Retrieved 2025-02-12 from https://catalog.data.gov/dataset/npl-superfund-site-boundaries-epa9.

United States Environmental Protection Agency (U.S. EPA). (2025b). "EPA Facility Registry Service (FRS): RMP." Dataset. Retrieved 2025-02-15 from https://catalog.data.gov/dataset/epa-facility-registry-service-frs-rmp5.

United States Environmental Protection Agency (U.S. EPA). (2025c). "Facility-level loadings output for 2024." Dataset. Retrieved 2025-02-12 from https://echo.epa.gov/trends/loading-tool/get-data/custom-search.

United States Geological Survey (USGS). (2023). "National Hydrography Dataset." Dataset. Retrieved 2023-09-13 from https://www.epa.gov/waterdata/get-nhdplus-national-hydrography-dataset-plus-data.
